# Translation and Validation of the Boston Technical Performance Score in a Developing Country

**DOI:** 10.21470/1678-9741-2021-0485

**Published:** 2021

**Authors:** Leonardo A. Miana, Meena Nathan, Davi Freitas Tenório, Valdano Manuel, Gustavo Guerreiro, Natália Fernandes, Carolina Vieira de Campos, Paula V. Gaiolla, Renata Sá Cassar, Aida Turquetto, Luciana Amato, Luiz Fernando Canêo, Larissa Leitão Daroda, Marcelo Biscegli Jatene, Fabio B. Jatene

**Affiliations:** 1 Pediatric Cardiology and Cardiovascular Surgery Division, Instituto do Coração do Hospital das Clínicas da Faculdade de Medicina da Universidade de São Paulo (InCor-HCFMUSP), Universidade de São Paulo, São Paulo, São Paulo, Brazil.; 2 Department of Cardiac Surgery, Boston Children's Hospital and Harvard Medical School, Boston, Massachusetts, United States of America.; 3 Cardiovascular Surgery Division, Clínica Girassol, Luanda, Angola.; 4 Surgery Department, Universidade Federal de Juiz de Fora, Minas Gerais, Brazil.

**Keywords:** Cardiac Surgical Procedures, Risk Adjustment, Congenital Heart Surgery, Hospital Mortality, Postoperative Period, Reference Standards

## Abstract

**Introduction:**

The Technical Performance Score (TPS) was developed and subsequently refined at the Boston Children's Hospital. Our objective was to translate and validate its application in a developing country.

**Methods:**

The score was translated into the Portuguese language and approved by the TPS authors. Subsequently, we studied 1,030 surgeries from June 2018 to October 2020. TPS could not be assigned in 58 surgeries, and these were excluded. Surgical risk score was evaluated using Risk Adjustment in Congenital Heart Surgery (or RACHS-1). The impact of TPS on outcomes was studied using multivariable linear and logistic regression adjusting for important perioperative covariates.

**Results:**

Median age and weight were 2.2 (interquartile range [IQR] = 0.5-13) years and 10.8 (IQR = 5.6-40) kilograms, respectively. In-hospital mortality was 6.58% (n=64), and postoperative complications occurred in 19.7% (n=192) of the cases. TPS was categorized as 1 in 359 cases (37%), 2 in 464 (47.7%), and 3 in 149 (15.3%). Multivariable analysis identified TPS class 3 as a predictor of longer hospital stay (coefficient: 6.6; standard error: 2.2; *P*=0.003), higher number of complications (odds ratio [OR]: 1.84; 95% confidence interval [CI]: 1.1-3; *P*=0.01), and higher mortality (OR: 3.2; 95% CI: 1.4-7; *P*=0.004).

**Conclusion:**

TPS translated into the Portuguese language was validated and showed to be able to predict higher mortality, complication rate, and prolonged postoperative hospital stay in a high-volume Latin-American congenital heart surgery program. TPS is generalizable and can be used as an outcome assessment tool in resource diverse settings.

**Figure:**
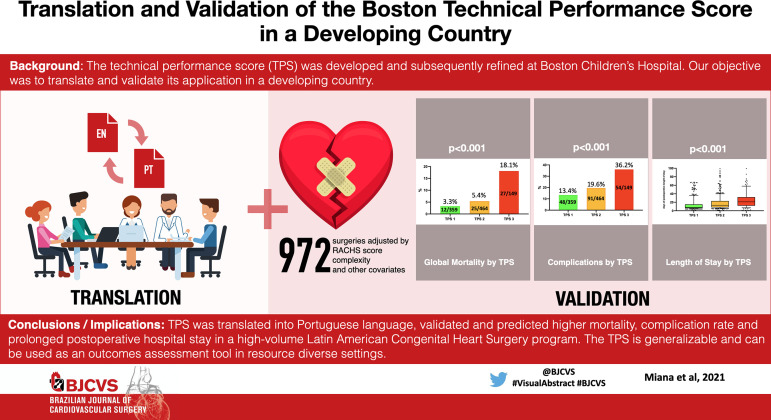

**Table:** 

Abbreviations, acronyms & symbols				
AUC	= Area under the curve		M/F	= Male/female
CHD	= Congenital heart disease		MV	= Mechanical ventilation
CI	= Confidence interval		NA	= Not assigned
CPB	= Cardiopulmonary bypass		OR	= Odds ratio
DSC	= Delayed sternal closure		RACHS-1	= Risk Adjustment in Congenital Heart Surgery
ECMO	= Extracorporeal membrane oxygenation		ROC	= Receiver operating characteristic
IQR	= Interquartile ranges		TPS	= Technical Performance Score
LOS	= Length of hospital stay		VIS	= Vasoactive inotropic score

## INTRODUCTION

Congenital heart disease (CHD) remains a major healthcare issue, and mitigating CHD mortality in developing countries could have a significant impact on the elevated childhood mortality rates^[1-3]^. As the vast majority of CHD require surgical treatment, improvement in surgical outcomes is highly desirable. Several factors affect the final outcome of CHD repair, and predictors of unfavorable events can often be identified before surgery^[4-6]^.

It is known that an optimal repair with trivial or no residual lesions results in better outcomes to the patient. However, it is not uncommon to find some degree of residual lesions after surgical repair, particularly after complex surgeries with multicomponent repairs^[7,8]^.

The big question is the acceptable level of residual lesion. Or yet, what is the real impact of these echocardiographic findings on the patient's outcomes? The Boston Children's Hospital developed a quality assessment tool, the Technical Performance Score (TPS)^[9-13]^, that quantified the degree of residual lesions based on echocardiographic and clinical findings.

Our objective was to translate TPS into the Portuguese language and to validate its utility and significance in a developing country with less predictable outcomes for CHD surgery^[14-17]^.

## METHODS

### Translation

We performed the translation using international protocols for the validation of scales and scores^[18]^. Two cardiac surgeons with a degree in English language made two separate versions of the TPS translated to Portuguese (LAM, DFT)^[19]^. Then, a panel of experts selected a final version. The third step consisted of a back-translation to the English language performed by a professional translator.

Finally, consensus Portuguese and back-translation versions were sent to the author (MN) for approval^[19]^. The final Portuguese TPS version is available as a supplement file.

### Validation

We included consecutive surgeries performed at a single institution between June 2018 and October 2020. Institutional approval with waiver of consent was obtained for this quality improvement initiative under the registration number 95807418.1.0000.0068. We collected demographic data and calculated the complexity risk score using the Risk Adjustment in Congenital Heart Surgery (RACHS-1) mortality categories^[20,21]^. Cardiopulmonary bypass (CPB) and aortic cross-clamping times, serum lactate levels at the end of surgery, vasoactive inotropic score (VIS) in the immediate postoperative period and in the first 24 hours following surgery, and TPS were assigned as outlined below. Measured outcomes were the incidence of postoperative complications, postoperative length of hospital stay (LOS), and mortality.

### Technical Performance Score

TPS was calculated based on the last postoperative echocardiogram available before discharge or death and other clinical and laboratory data as published in previous articles from the Boston group^[8,9-13,22]^. The TPS was calculated for the index operation of each hospitalization. Index operation is defined as the first congenital heart surgery performed either on CPB or off bypass.

### Vasoactive Inotropic Score

We calculated VIS when the patient arrived at the intensive care unit and then 24 hours later^[23,24]^.

### Postoperative Complications or Serious Adverse Events

We considered as postoperative complications the following major adverse events defined by the Society of Thoracic Surgeons Congenital Heart Surgery Database (or STS-CHSD): (1) cardiopulmonary arrest, (2) need for extracorporeal membrane oxygenation (ECMO), (3) re-exploration for hemodynamic instability, (4) re-exploration for bleeding or (5) mediastinitis, (6) diaphragm plication, (7) stroke, and (8) renal failure requiring dialysis^[25,26]^.

Reoperations or surgical and catheter based reintervention on the anatomic areas of repair were not considered as adverse events because they are components of TPS.

### Operative Mortality

Operative mortality was considered as death in the hospital or within 30 days of the operation if the patient was discharged before this period.

### Exclusion Criteria

Operations where TPS could not be calculated either due to lack of availability of postoperative echocardiogram or because the surgery performed does not have a TPS developed yet were excluded.

### Statistical Analysis

Data were described using medians and interquartile ranges (IQR) of 25 and 75% for continuous variables and absolute numbers and percentages for categorical variables.

We considered individual index surgeries rather than individual patients in our analyses because the same patient may have had more than one index surgery during the study's time span. For example, a patient who underwent Norwood procedure as a neonate, was discharged home, and four months later came back for a programed bidirectional Glenn operation was computed as two surgeries. However, if the patient had more than one surgical procedure in the same hospital stay, only the index surgery was considered.

The primary outcome was operative mortality. Secondary outcomes included postoperative complications and postoperative LOS. Other covariates included in adjusted analyses consisted of perioperative variables such as age, weight, case complexity as defined by RACHS-1 mortality categories, VIS and serum lactate at the end of surgery, and VIS 24 hours after surgery.

To study the associations between categorical outcome variables (mortality and postoperative complications) and explanatory variables, simple and multivariable logistic regression models were used. To evaluate the multivariable models, C statistic and receiver operating characteristic curve were used.

To analyze the associations between the continuous outcome (postoperative LOS) and the explanatory variables, simple and multivariable linear regression models were used.

The level of significance adopted in the tests was 0.05. Two-tailed hypotheses were considered. The software R (version 3.6.0) was used to carry out all the analyzes.

## RESULTS

**Fig. 1:**
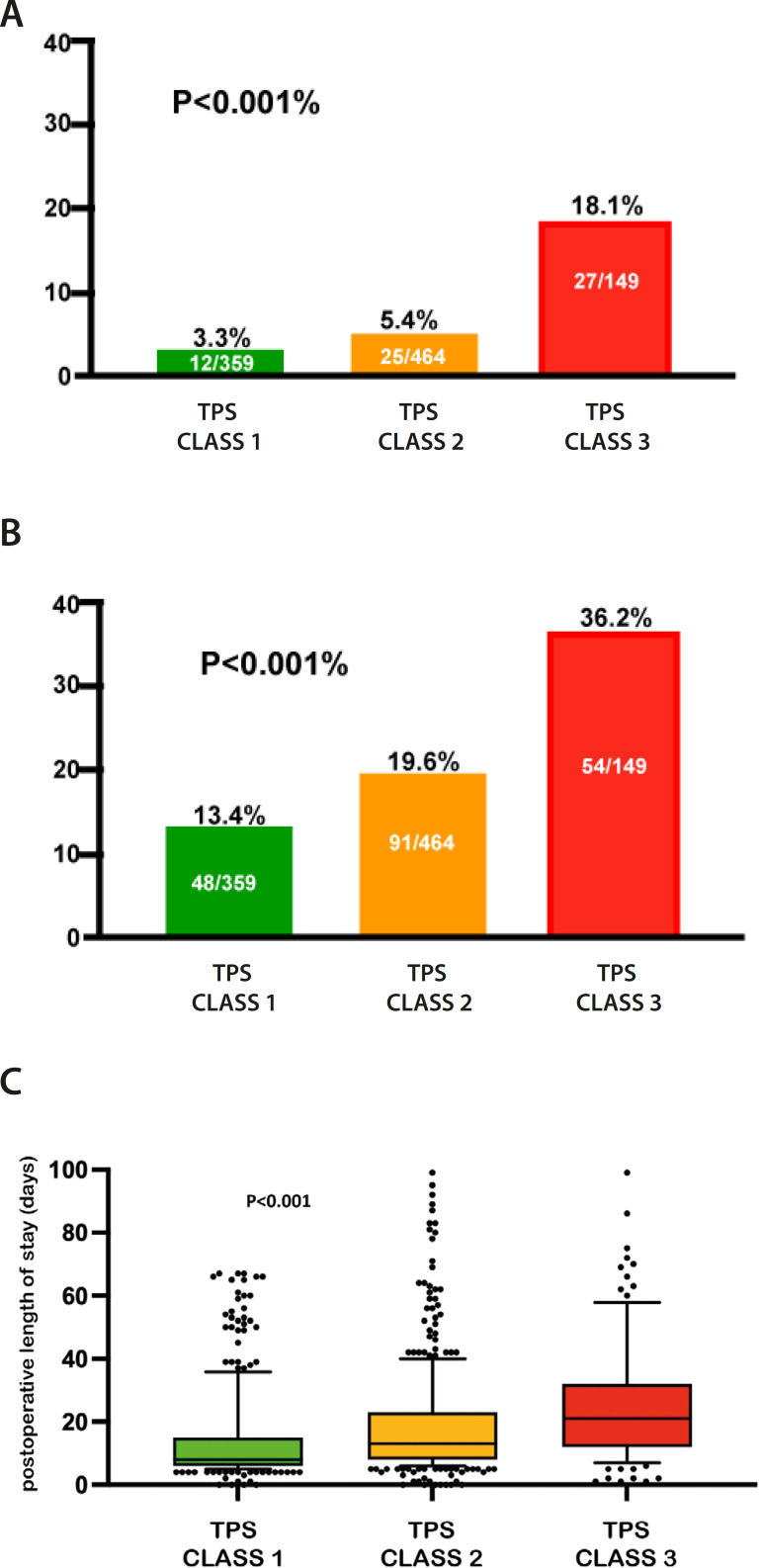
Outcomes measured by the Technical Performance Score (TPS). A) Global mortality according to the surgical result measured by TPS. B) Complications according to the surgical result measured by TPS. C) Graph of dispersion of the postoperative length of stay according to the surgical performance calculated by TPS.

A total of 1,030 consecutive eligible index surgeries between June 2018 and October 2020 were analyzed, 58 surgeries were subsequently excluded because TPS could not be assigned. Median age of the 972 patients was 2.2 (IQR = 0.5-13) years, and median weight was 10.8 (IQR = 5.6-40) kilograms (kg). Overall operative mortality was 6.58% (n=64), and postoperative complications occurred in 192 (19.7%) cases. Demographic distribution and overall outcomes are described in Table 1. Mortality in patients with TPS class 3 was statistically higher than in those with TPS classes 1 and 2 (TPS class 3 = 18.1%, TPS class 2 = 5.4%, and TPS class 1 = 3.3%; *P*<0.001). The distribution of mortality according to TPS is shown in Figure 1A and Table 2.

**Table 1.** Demographics, patients’ characteristics, and outcomes of interest.

Variable		N (%)/median (IQR)
Gender M/F		462/510 (47.5%/52.5%)
Age (days)		799 (210-4,742)
Weight (kg)		10.8 (5.6-40)
Previous surgery		259 (27.9%)
Chromosomal anomalies		147 (15.5%)
Age group	Neonate	58 (6%)
	Infant	304 (31.3%)
	Children	419 (43.1%)
	Adult	191 (19.6%)
RACHS-1	1	203 (20.9%)
	2	253 (26.1%)
	3	421 (43.4%)
	4	54 (5.6%)
	5-6	14 (1.4%)
	Not assigned	25 (2.6%)
Surgery with CPB		897 (92.3%)
CPB time (minutes)		101 (71-143)
Aortic cross-clamping time (minutes)		67 (37-100)
Serum lactate level (mg/dL)		22 (16-33.8)
VIS at the end of surgery		7.5 (3.7-12)
VIS after 24 hours		7.5 (1.6-14.5)
Delayed sternal closure		153 (15.8%)
ECMO need		27 (2.8%)
TPS	Class 1	359 (37%)
	Class 2	464 (47.7%)
	Class 3	149 (15.3%)
Postoperative complications		192 (19.7%)
Unplanned reintervention		40 (4.1%)
Early death		64 (6.58%)
Postoperative LOS (days)		11 (7-22)

CPB=cardiopulmonary bypass; ECMO=extracorporeal membrane oxygenation; IQR=interquartile ranges; LOS=length of hospital stay; M/F=male/female; RACHS-1=Risk Adjustment in Congenital Heart Surgery; TPS=Technical Performance Score; VIS=vasoactive inotropic score

**Table 2.** Distribution of primary endpoints according to TPS and RACHS-1.

Variable		Distribution	OR or coefficient	95% confidence intervals or standard error	*P*-value
		N (%) or median (IQR)			
**Mortality**					
TPS	Class 1	12 (3.3%)	Reference	Reference	Reference
	Class 2	25 (5.4%)	1.5	0.8-3	0.24
	Class 3	27 (18.1%)	5.9	2.9-11.7	< 0.001
RACHS-1	Category 1	0 (0%)	Reference	Reference	Reference
	Category 2	9 (3.5%)	7.5	0.9-59.2	0.06
	Category 3	41 (9.7%)	21.8	3-159.3	0.002
	Category 4	4 (7.4%)	16.2	1.8-147.6	0.014
	Category 5-6	7 (50%)	202	21.8-1869.7	< 0.001
	Not assigned	3 (12%)	27.6	2.8-275.9	0.005
**Complications**					
TPS	Class 1	48 (13.4%)	Reference	Reference	Reference
	Class 2	91 (19.6%)	1.6	1.1-2.3	0.024
	Class 3	54 (36.2%)	3.7	2.3-5.8	< 0.001
RACHS-1	Category 1	9 (4.4%)	Reference	Reference	Reference
	Category 2	39 (15.4%)	3.9	1.9-8.3	< 0.001
	Category 3	108 (25.6%)	7.4	3.6-14.9	< 0.001
	Category 4	19 (35.2%)	11.7	5/28	< 0.001
	Category 5-6	12 (85.7%)	129.3	25.1-666.3	< 0.001
	Not assigned	6 (24%)	6.8	2.2-21.2	< 0.001
**Postoperative length of hospital stay**					
TPS	Class 1	8 (6-14.2)	Reference	Reference	Reference
	Class 2	13 (8-23)	5.9	1.6	< 0.001
	Class 3	21 (12-31.7)	14.3	2.4	< 0.001
RACHS-1	Category 1	7 (5.8-8)	Reference	Reference	Reference
	Category 2	11 (7-18)	6.1	2.0	0.003
	Category 3	16 (8-26)	13.8	1.9	< 0.001
	Category 4	22 (13-38)	26.8	3.4	< 0.001
	Category 5-6	21 (15-37)	35.9	8.2	< 0.001
	Not assigned	54 (41-60)	39.6	4.9	< 0.001

IQR=interquartile ranges; OR=odds ratio; RACHS=Risk Adjustment in Congenital Heart Surgery; TPS=Technical Performance Score

Similarly, more complications were observed in patients with TPS class 3 (TPS class 3 = 36.2%, TPS class 2 = 19.6%, and TPS class 1 = 13.4%; *P*<0.001) (Figure 1B and Table 2).

Postoperative LOS was significantly longer in patients with TPS class 3 (median = 21, IQR = 12-31 days) compared to patients with TPS class 2 (median = 13, IQR = 8-23 days) or TPS class 1 (median = 8, IQR = 6-14.2 days) (Figure 1C and Table 2) (*P* < 0.001).

Aditionally, patients with higher RACHS-1 mortality risk had a higher chance of death, complications, and LOS. Likewise, a higher incidence of inadequate performance (TPS class 3) was observed in patients with higher RACHS-1 mortality category (Tables 2 and 3).

**Table 3.** Variables stratified by Technical Performance Score (TPS) classification.

		TPS class 1 N (%) or median (IQR) N=359	TPS class 2 N (%) or median (IQR) N=464	TPS class 3 N (%) or median (IQR) N=149	Univariate *P*-value
Age (days)		1360 (317-5663)	684 (196-4827)	328 (136-1846)	0.006
Weight (kilograms)		13.8 (7-49)	10 (5.5-41)	7.4 (4.6-17)	0.002
Age group	Neonate	16 (4.5%)	30 (6.5%)	11 (7.4%)	< 0.001
	Infant	83 (23.1%)	152 (32.8%)	70 (47%)	
	Children	179 (49.9%)	192 (41.4%)	51 (34.2%)	
	Adult	84 (23.4%)	90 (19.4%)	17 (11.4%)	
RACHS-1	Category 1	172 (47.9%)	32 (6.9%)	1 (0.7%)	< 0.001
	Category 2	76 (21.2%)	142 (30.6%)	35 (23.5%)	
	Category 3	85 (23.7%)	242 (52.1%)	94 (63%)	
	Category 4	7 (1.9%)	33 (7.1%)	14 (9.4%)	
	Categories 5-6	5 (1.4%)	5 (1.1%)	4 (2.7%)	
	Not assigned	14 (3.9%)	10 (2.2%)	1 (0.7%)	
CPB time (minutes)		80 (57-101)	117 (90-150)	150 (115-181)	< 0.001
Aortic cross-clamping time (minutes)		41 (24-64)	80 (51-106)	100 (70-123)	< 0.001
Serum lactate level (mg/dL)		21 (14-30)	24 (17-35)	23 (18-39)	< 0.001
VIS at the end of surgery		5 (2-9)	8 (5-13)	10.7 (7.5-17.6)	< 0.001
VIS after 24 hours		3.7 (0-8)	8.5 (3-15.5)	14 (8-24.5)	< 0.001
MV (hours)		6 (3.7-24)	19.5 (5-96)	27.5 (8-162)	< 0.001
DSC		24 (6.7%)	80 (17.2%)	49 (32.9%)	< 0.001
Complications		48 (13.4%)	91 (19.6%)	54 (36%)	< 0.001
Postoperative LOS		8 (6-14.2)	13 (8-23)	21 (12-31.7)	< 0.001
Mortality		12 (3.3%)	25 (5.4%)	27 (18.1%)	< 0.001
Days on inotropes		2 (1-3)	3 (1-8)	6 (2-12)	< 0.001

CPB=cardiopulmonary bypass; DSC=delayed sternal closure; IQR=interquartile ranges; LOS=length of hospital stay; MV=mechanical ventilation; RACHS=Risk Adjustment in Congenital Heart Surgery; VIS=vasoactive inotropic score

Multivariable analysis to determine the association between TPS and outcomes adjusted for important perioperative variables including case complexity determined that TPS class 3 was associated with a 1.8 times greater chance of postoperative complications, and 3.2 times greater chance of death compared to TPS class 1. There was an average of 6.6 more days of hospitalization in the postoperative period in surgeries classified as TPS class 3. On the other hand, outcomes of surgeries assigned as TPS class 2 were not statistically different from those assigned as TPS 1 (Tables 4, 5, and 6).

**Table 4.** Mortality risk factors — multivariable analysis.

Variable	Coefficient	Standard error coefficient	Odds ratio	95% confidence interval	Multivariable
					*P*-value
TPS 2	0.09	0.4	1.1	0.5-2.4	0.81
TPS 3	1.16	0.4	3.2	1.4-7	0.004
RACHS-1 2	1.29	1.1	3.6	0.4-30.5	0.23
RACHS-1 3	2.25	1.1	9.5	1.2-73.7	0.032
RACHS-1 4	1.14	1.2	3.1	0.3-32.7	0.34
RACHS-1 6	3.5	1.2	33.2	3.2-340.9	0.003
RACHS-1 NA	3.01	1.2	20.3	1.9-217.5	0.013
Age (years)	0.12	0.04	1.1	1-1.22	0.004
Weight (kilograms)	-0.12	0.03	0.9	0.8-0.9	< 0.001
Serum lactate level (mg/dL)	0.02	0.01	1	1.01-1.04	0.001

NA=not assigned; RACHS-1=Risk Adjustment in Congenital Heart Surgery; TPS=Technical Performance Score

**Table 5.** Postoperative complication risk factors — multivariable analysis.

Variable	Coefficient	Standard error coefficient	Odds ratio	95% confidence interval	Multivariable
					*P*-value
TPS 2	- 0.03	0.22	1	0.6-1.5	0.88
TPS 3	0.61	0.26	1.8	1.1-3.1	0.019
RACHS-1 2	1.04	0.41	2.8	1.3-6.3	0.011
RACHS-1 3	1.63	0.39	5.1	2.4-10.9	< 0.001
RACHS-1 4	1.82	0.49	6.2	2.4-16.1	< 0.001
RACHS-1 6	4.04	0.86	56.7	10.4-308.4	< 0.001
RACHS-1 NA	1.60	0.59	5	1.6-15.9	0.007
Weight (kilograms)	-0.02	0.00	0.98	0.98-0.99	< 0.001
Serum lactate level (mg/dL)	0.01	0.005	1.01	1-1.02	0.014

NA=not assigned; RACHS-1=Risk Adjustment in Congenital Heart Surgery; TPS=Technical Performance Score

**Table 6.** Postoperative length of stay risk factors — multivariable analysis.

Variable	Coefficient	Standard error coefficient	Multivariable
			*P*-value
TPS 2	1.87	1.64	0.25
TPS 3	6.65	2.21	0.003
RACHS-1 2	2.68	2.18	0.22
RACHS-1 3	9.15	2.08	< 0.001
RACHS-1 4	18.99	3.46	< 0.001
RACHS-1 6	15.17	5.87	0.01
RACHS-1 NA	30.79	4.43	< 0.001
Age (years)	-0.15	0.05	0.003
Serum lactate level (mg/dL)	0.11	0.04	0.006

Class 1 TPS and RACHS-1 mortality category 1 were used as reference category

NA=not assigned; RACHS-1=Risk Adjustment in Congenital Heart Surgery; TPS=Technical Performance Score

**Fig. 2:**
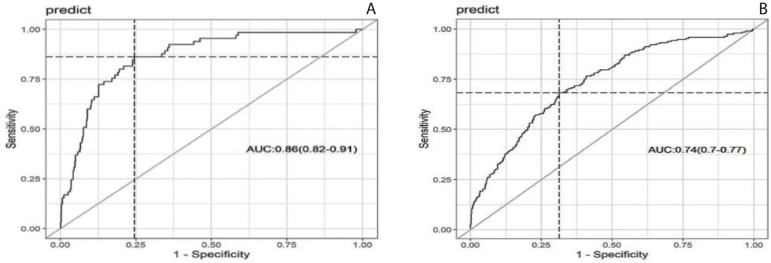
Multivariate model analysis through the receiver operating characteristic (ROC) curve. A) Mortality multivariate model analysis through the ROC curve. B) Complications multivariate model analysis through the ROC curve. AUC=area under the curve.

Assessment of model performance by C statistic confirmed that multivariable logistic models had good discrimination with area under the curve (AUC) for mortality of 0.86 (95% CI = 0.82-0.91) and for complications of 0.74 (95% CI = 0.70-0.77) (Figure 2). For postoperative LOS, linear regression R2 was 0.14.

## DISCUSSION

Outcomes after congenital heart surgery are consequences of multiple variables. Therefore, it becomes challenging to predict the impact of surgical correction itself on the final result^[27]^.

Since 2018, we have been using TPS as part of our congenital cardiac surgical team's performance analysis. Our findings in 972 surgeries strengthens the role of TPS as a predictor of adverse outcomes after adjusting for other covariates. Patients assigned as an inadequate surgical result had 3.2 times more chance of death and 1.8 more chances of complication.

Our multivariable models presented a significant AUC (0.86 for mortality and 0.74 for complications), even including perioperative variables, some of which collinear with TPS (CPB time, VIS, and lactate levels). Patients with TPS class 3 who were discharged home stayed in hospital an average of six more days when compared to patients whose surgery was assigned as TPS classes 1 and 2. This demonstrates that pursuing a more technically adequate surgery, in addition to saving lives, may decrease resource utilization, leading to cost savings and the possibility of operating on more patients.

Since 2007, researchers at the Boston Children's Hospital have been demonstrating that TPS is an important predictor of short and medium-term outcomes^[6,9-13,22,28]^. Researchers from England and Japan also evaluated their surgical performance and postoperative outcomes using TPS with similar findings^[29,30]^.

Therefore, the importance of translating TPS into Portuguese and its routine use and validation in our environment was key to increase the awareness of this tool among our surgical community. Our expectation is that this will also allow comparison and evaluation of surgical performance in smaller programs with erratic outcomes, helping them identify opportunities for improvement.

Nevertheless, we observed considerably higher mortality and complication rates in surgeries with TPS assigned as classes 1 and 2 than have been previously reported in developed countries, suggesting that TPS explains some, but not all of the differences in outcomes across the globe^[22,31]^.

Jacques et al.^[32]^ previously demonstrated that, in their experience, postoperative errors had more impact on mortality than technical issues. We are aware that TPS, which measures the adequacy of surgical repair, cannot be considered in isolation as a score that can predict outcomes perfectly. But it has been shown to be a key component of perioperative course prediction.

### Limitations

This is a retrospective single-center study with its inherent limitation related to missing data, inaccuracy, and incompleteness in documentation of events. Generalizability of this study's findings may be limited to centers with a similar case mix and resource distribution. We also included factors such as CPB times, aortic cross-clamping times, VIS, and lactate levels that are known to be collinear with TPS and thus may have diluted the effects of the covariates in the model. However, despite these limitations, this study provides outcome analyses on a large sample from the biggest Latin-American cardiology center.

## CONCLUSION

TPS class 3 was a predictor of higher mortality and complications with consequent longer postoperative LOS in a developing country high-volume congenital cardiovascular surgery program. The present study further corroborates the validity of TPS as a predictor of postoperative outcomes. The translation of TPS associated with its guidelines may help expand adoption in our environment and assist more programs in targeting improvements in outcomes.

**Table:** 

Authors' roles & responsibilities	
LAM	Substantial contributions to the conception or design of the work; or the acquisition, analysis, or interpretation of data for the work; drafting the work or revising it critically for important intellectual content; agreement to be accountable for all aspects of the work in ensuring that questions related to the accuracy or integrity of any part of the work are appropriately investigated and resolved; final approval of the version to be published
MN	Drafting the work or revising it critically for important intellectual content; agreement to be accountable for all aspects of the work in ensuring that questions related to the accuracy or integrity of any part of the work are appropriately investigated and resolved; final approval of the version to be published
DFT	Substantial contributions to the conception or design of the work; or the acquisition, analysis, or interpretation of data for the work
VM	Substantial contributions to the conception or design of the work; or the acquisition, analysis, or interpretation of data for the work
GG	Substantial contributions to the conception or design of the work; or the acquisition, analysis, or interpretation of data for the work
NF	Substantial contributions to the conception or design of the work; or the acquisition, analysis, or interpretation of data for the work
CVC	Substantial contributions to the conception or design of the work; or the acquisition, analysis, or interpretation of data for the work
PVG	Substantial contributions to the conception or design of the work; or the acquisition, analysis, or interpretation of data for the work
RSC	Substantial contributions to the conception or design of the work; or the acquisition, analysis, or interpretation of data for the work
AT	Agreement to be accountable for all aspects of the work in ensuring that questions related to the accuracy or integrity of any part of the work are appropriately investigated and resolved; final approval of the version to be published
LA	Agreement to be accountable for all aspects of the work in ensuring that questions related to the accuracy or integrity of any part of the work are appropriately investigated and resolved; final approval of the version to be published
LFC	Agreement to be accountable for all aspects of the work in ensuring that questions related to the accuracy or integrity of any part of the work are appropriately investigated and resolved; final approval of the version to be published
LLD	Agreement to be accountable for all aspects of the work in ensuring that questions related to the accuracy or integrity of any part of the work are appropriately investigated and resolved; final approval of the version to be published
MBJ	Agreement to be accountable for all aspects of the work in ensuring that questions related to the accuracy or integrity of any part of the work are appropriately investigated and resolved; final approval of the version to be published
FBJ	Agreement to be accountable for all aspects of the work in ensuring that questions related to the accuracy or integrity of any part of the work are appropriately investigated and resolved; final approval of the version to be published

## References

[1] Malta DC, Duarte EC, Escalante JJ (2010). Avoidable causes of infant mortality in Brazil, 1997-2006: contributions to performance evaluation of the Unified National Health System. Cad Saude Publica.

[2] BRASIL (2017). Plano Nacional de Assistência à Criança com Cardiopatia Congênita.

[3] Pinto VC Jr, Fraga Mde N, Freitas SM, Croti UA (2013). Regionalization of Brazilian pediatric cardiovascular surgery. Rev Bras Cir Cardiovasc.

[4] Barach P, Johnson JK, Ahmad A (2008). A prospective observational study of human factors, adverse events, and patient outcomes in surgery for pediatric cardiac disease. J Thorac Cardiovasc Surg.

[5] de Leval MR, Carthey J, Wright DJ (2000). Human factors and cardiac surgery: a multicenter study. J Thorac Cardiovasc Surg.

[6] Nathan M, Karamichalis JM, Liu H (2011). Intraoperative adverse events can be compensated by technical performance in neonates and infants after cardiac surgery: A prospective study. J Thorac Cardiovasc Surg.

[7] Karamichalis JM, Thiagarajan RR, Liu H (2010). Stage I Norwood: Optimal technical performance improves outcomes irrespective of preoperative physiologic status or case complexity. J Thorac Cardiovasc Surg.

[8] Larrazabal LA, del Nido PJ, Jenkins KJ (2007). Measurement of technical performance in congenital heart surgery: a pilot study. Ann Thorac Surg.

[9] Nathan M, Sleeper LA, Ohye RG (2014). Technical Performance Score is Associated with Outcomes after the Norwood Procedure. J Thorac Cardiovasc Surg.

[10] Nathan M, Gauvreau K, Samnaliev M (2016). Technical Performance Score Predicts Resource Utilization in Congenital Cardiac Procedures. J Am Coll Cardiol.

[11] Nathan M, Pigula FA, Liu H (2013). Inadequate Technical Performance Scores Are Associated With Late Mortality and Late Reintervention. Ann Thorac Surg.

[12] Nathan M, Karamichalis J, Liu H (2014). Technical Performance Scores are strongly associated with early mortality, postoperative adverse events and ICU length of stay - Analysis of consecutive discharges for 2 years. J Thorac Cardiovasc Surg.

[13] Nathan M, Sadhwani A, Gauvreau K (2014). Association between Technical Performance Scores and neurodevelopmental outcomes after congenital cardiac surgery. J Thorac Cardiovasc Surg.

[14] Carmona F, Manso PH, Ferreira MN (2017). Collaborative Quality Improvement in the Congenital Heart Defects Development of the ASSIST Consortium and a Preliminary Surgical Outcomes Report. Braz J Cardiovasc Surg.

[15] Vilela R, Haickel DA, Elinor M (2007). Is the RACHS-1 (Risk adjustment in congenital heart surgery) a useful tool in our scenario?. Braz J Cardiovasc Surg.

[16] Rocha LA, Froio SC, Silva CC, Negreiros SDA (2018). Risk Factors for Mortality in Children with Congenital Heart Disease Delivered at a Brazilian Tertiary Center. Braz J Cardiovasc Surg.

[17] Miana LA, Manuel V, Turquetto AL (2020). Atrioventricular Valve Repair in Single Ventricle Physiology: Timing Matters. World J Pediatr Congenit Heart Surg.

[18] Beaton DE, Bombardier C, Guillemin F (2000). Guidelines for the Process of Cross-Cultural Adaptation of Self- Report Measures. Spine.

[19] Nathan N, Karamichalis J (2015). Sabiston & Spencer surgery of the chest.

[20] Jenkins KJ, Gauvreau K, Newburger JW (2002). Consensus-based method for risk adjustment for surgery for congenital heart disease. J Thorac Cardiovasc Surg.

[21] Jenkins KJ (2004). Risk adjustment for congenital heart surgery: the RACHS-1 method. Semin Thorac Cardiovasc Surg Pediatr Card Surg Annu.

[22] Nathan M, Karamichalis JM, Liu H (2012). Surgical technical performance scores are predictors of late mortality and unplanned reinterventions in infants after cardiac surgery. J Thorac Cardiovasc Surg.

[23] Gaies MG, Jeffries HE, Niebler RA (2014). Vasoactive-inotropic score is associated with outcome after infant cardiac surgery: an analysis from the Pediatric Cardiac Critical Care Consortium and Virtual PICU System Registries. Pediatr Crit Care Med.

[24] Talwar S, Bansal A, Sahu MK (2018). Vasoactive Inotropic Score and Outcome Assessment in Cyanotic Infants after Cardiovascular Surgery. J Card Crit Care.

[25] Jacobs JP, Mavroudis C, Jacobs ML, STS Congenital Database Taskforce, Joint EACTS-STS Congenital Database Committee (2006). What is operative mortality? Defining death in a surgical registry database: a report of the STS Congenital Database Taskforce and the Joint EACTS-STS Congenital Database Committee. Ann Thorac Surg.

[26] Jacobs JP, Benavidez OJ, Bacha EA (2008). The nomenclature of safety and quality of care for patients with congenital cardiac disease: a report of the Society of Thoracic Surgeons Congenital Database Taskforce Subcommittee on Patient Safety. Cardiol Young.

[27] Agarwal HS, Wolfram KB, Saville BR (2014). Postoperative complications and association with outcomes in pediatric cardiac surgery. J Thorac Cardiovasc Surg.

[28] Shuhaiber J, Gauvreau K, Thiagarjan R (2012). Congenital heart surgeon's technical proficiency affects neonatal hospital survival. J Thorac Cardiovasc Surg.

[29] Bellsham-Revell HR, Deri A, Caroli S (2019). Application of the Boston Technical Performance Score to intraoperative echocardiography. Echo Res Pract.

[30] Shimada M, Hoashi T, Iida J, Ichikawa H (2020). The Impact of Post-Graduate Year of Primary Surgeon on Technical Performance Score in Tetralogy of Fallot Repair.

[31] Pasquali SK, He X, Jacobs JP (2012). Evaluation of Failure to Rescue as a Quality Metric in Pediatric Heart Surgery: An Analysis of The STS Congenital Heart Surgery Database. Ann Thorac Surg.

[32] Jacques F Anand V, Hickey EJ A (2013). Medical errors: The performance gap in hypoplastic left heart syndrome and physiologic equivalents?.

