# Supplementary material for: Translation and Validation of the Boston Technical Performance Score in a Developing Country
Source: Braz J Cardiovasc Surg. 2021 Sep-Oct;36(5):589–98. doi: 10.21470/1678-9741-2021-0485 (PMC8597612; doi:10.21470/1678-9741-2021-0485)
Supplement: Supplementary file 1 [file rbccv-36-05-0589-suppl01.pdf]

SUPPLEMENT

DOI: 10.21470/1678-9741-2021-0485s

ESCORE DE PERFORMANCE TÉCNICA (EPT)

TECHNICAL PERFORMANCE SCORE (TPS)

DIRETRIZES GERAIS/CRITÉRIOS

Três categorias de Escore de Performance Técnica (EPT):

- Classe 1 (nenhuma ou mínima lesão residual; ótimo);
- Classe 2 (lesão residual menor; adequado);
- Classe 3 (lesão residual maior ou lesão residual que requer reintervenção; inadequado).

CRITÉRIOS DE PONTUAÇÃO

1. Reintervenção pós-operatória em áreas anatômicas inicialmente operadas:
   A. Reintervenção cirúrgica
   B. Reintervenção por cateterismo
2. Dados do Ecocardiograma (pré-alta ou pré-intervenção).
3. Dados clínicos como gradientes pressóricos, necessidade inesperada de marcapasso permanente e etc. Dados clínicos serão usados quando outros dados estiverem insuficientes ou indisponíveis.

Cada operação é dividida em subprocedimentos separados, os quais são pontuados individualmente.

Se TODOS subprocedimentos são Classe 1, então o escore global é Classe 1.

Se QUALQUER subprocedimento é Classe 2 e nenhum é Classe 3, então o escore global é Classe 2.

Se QUALQUER subprocedimento é Classe 3, então o escore global é Classe 3.

Apenas as áreas anatômicas reparadas são pontuadas. Por exemplo, em um paciente com múltiplas CIVs, no qual certas CIVs são intencionalmente deixadas abertas, o escore total da operação não é influenciado pela presença destas CIVs não abordadas. De forma semelhante, no Truncus Arteriosus, a valva truncal com insuficiência discreta que não é abordada é desconsiderada do sistema de pontuação.

Complicações técnicas inesperadas em áreas anatômicas nas quais não se pretendia operar, por exemplo, uma lesão acidental de grandes vasos, coronárias, ventrículos ou átrios, farão parte do escore de performance técnica. Por exemplo, será parte de um subprocedimento que receberá uma pontuação como parte de toda a operação.

Reintervenções/revisões pós-operatórias em sala de cateterismo cardíaco ou centro cirúrgico na área anatômica operada rebaixam o escore global para Classe 3.

ESCORE DE PERFORMANCE TÉCNICA (LESÃO RESIDUAL) SIMPLIFICADO

1. Identificar e listar os componentes do procedimento cardíaco executado.
2. Houve reintervenção pós-operatória (por cateterismo ou cirúrgica) executada na área anatômica operada devido a deficiências técnicas (excluindo reabertura do tórax por baixo débito, reoperações por sangramento e etc.)?
   - **Sim** - escore técnico = Classe 3 - Inadequado
   - **Não** - prosseguir para:
     A. Análise do ecocardiograma pré-alta procurando por:
        a.1. Defeitos residuais
        a.2. Gradientes ou insuficiências residuais (para valvas)
        a.3. Anomalias anatômicas residuais com consequências hemodinâmicas (ex.: Acotovelamento em Shunt)
     B. Evolução clínica
        b.1. Avaliar necessidade de marcapasso permanente antes da alta
        Para Neonatos: Ecocardiograma completo após extubação e estabilidade hemodinâmica antes da transferência da UTI devem ser considerados para pontuação ao invés do Ecocardiograma pré-alta, uma vez que estes pacientes podem ter internações superiores a um mês.
        Para pacientes com internações de longa duração em UTI (ex.: dispositivos de assistência ventricular, transplantes e etc): Ecocardiograma completo executado entre o 7º dia e o 14º dia de pós-operatório deve ser usado para pontuação.
3. Listar o escore global e marcar escores inadequados com nota explanatória.

TABELA DE CONTEÚDO:

Módulos

1. Fechamento de PCA, cirúrgico - pág. 2
2. Correção de CIA (Atriosseptoplastia, Atriosseptorrafia, Átrio Único) - pág. 2
3. Correção de Conexão Anômala Parcial de Veias Pulmonares, incluindo Síndrome de Cimitarra - pág. 3
4. Correção de CIA tipo Seio Venoso - pág. 3
5. Correção de CIV (Ventriculosseptoplastia, Ventriculosseptorrafia, CIVs múltiplas) - pág. 4
6. Correção de DSAV (Parcial, Transicional, Total) - pág. 5
7. Correção de T4F (+/- ventriculotomia, +/- remendo transanular, preservação da valva pulmonar) - pág. 6

8. Correção de CoAo (término-terminal, término-terminal extendida, +/- plastia com retalho ou remendo, +/- interposição de enxerto) - pág. 7
9. Operação de Jatene (+ variações de D-TGA/septo interventricular intacto, D-TGA/CIV, D-TGA/+/- CIV/anomalia do arco aórtico, Taussig-Bing) - pág. 8
10. Correção de Conexão Anômala Total de Veias Pulmonares - CATVP (supra-cardíaca, intracardíaca, infra-cardíaca, mista) - pág. 9
11. Correção da Interrupção do Arco Aórtico (IAAo) +/- CIV +/- CIA, Coarctação + CIV, Arco áortico +/- CIV - pág. 10
12. Correção de Truncus Arteriosus (+-Interrupção do Arco Aórtico) - pág. 11
13. Shunt sistêmico-pulmonar, Operação de Blalock-Taussig Modificada - pág. 12
14. Operação de Norwood - pág. 12
15. Operação de Glenn (anastomose cavo-pulmonar uni- ou bidirecional; Glenn uni- ou bidirecional, +/- Reconstrução de artérias pulmonares) - pág. 13
16. Operação de Fontan, Conexão cavo-pulmonar total - CCPT (túnel lateral, +/- fenestração) - pág. 13
17. Operação de Fontan, Conexão cavo-pulmonar total - CCPT (tubo extracardíaco, +/- fenestração) - pág. 13
18. Correção da Estenose Aórtica +/- Subvalvar +/- Supravalvar, +/- Valvar - pág. 14
19. Troca da Valvar Aórtica, incluindo Valva Truncal, Prótese mecânica, Prótese Biológica - pág. 15
20. Operação de Ross, Ross-Konno, Konno modificado - pág. 15
21. Substituição da Raiz Aórtica, preservando a valva aórtica, homoenxerto - pág. 16
22. Valvoplastia Mitral para Estenose ou Insuficiência, Anel supravalvar mitral, Substituição Valvar Mitral, Prótese Biológica, Prótese Mecânica - pág. 16
23. Valvoplastia Aórtica, Valva Truncal, para Estenose ou Insuficiência - pág. 17
24. Valvoplastia Tricúspide - pág. 17
25. Substituição da Valva Tricúspide - pág. 18
26. Correção da Anomalia de Ebstein/Técnica do Cone - pág. 18
27. Troca da Valva Pulmonar/Correção da obstrução da Via de Saída do Ventrículo Direito, Tubo VD-APs +/- TP, Plastia de artérias pulmonares, reconstrução (plastia) do TP, reoperação de tubo VD-APs - pág. 19
28. Correção Biventricular da SHCE - pág. 20
29. Transplante Cardíaco - pág. 21
30. Dispositivos de Assistência Circulatória Mecânica - pág. 21

GRUPO 1

Tratamento Cirúrgico da Persistência do Canal Arterial (PCA)

Subprocedimentos	Classe 1	Classe 2	Classe 3
Ligadura/Secção PCA	- Sem shunt residual	- Shunt residual < 1mm	- Reintervenção<br>- Shunt > 1mm

Correção de Comunicação Interatrial - CIA (patch/rafia primária, átrio único)

Subprocedimentos	Classe 1	Classe 2	Classe 3
Correção da CIA	- Sem shunt ou shunt mínimo:<br>. < 2mm se > 10kg<br>. < 1mm se < 10kg	- Shunt residual pequeno:<br>. 2-3mm se > 10kg<br>. 1-2mm se < 10kg	- Reintervenção<br>- Shunt residual moderado ou grande:<br>. > 3mm se > 10kg<br>. > 2mm se < 10kg
Sistema de Condução	- Condução Normal<br>- Sem mudanças em relação ao pré-op.	- Condução Normal<br>- Sem mudanças em relação ao pré-op.	Necessidade de marcapasso definitivo

Quando existe a intenção de fechamento parcial da CIA, a correção da CIA não é pontuada.

Correção de Conexão Anômala Parcial de Veias Pulmonares (CAPVP/DAPVP), incluindo Síndrome de Cimitarra
(Atenção: Quando usado gradiente em território venoso, deve-se levar em consideração o padrão de fluxo, uma vez que o gradiente é fluxo-dependente.)

**Subprocedimentos**	**Classe 1**	**Classe 2**	**Classe 3**
Veia Pulmonar	- Nenhuma obstrução<br>- Obstrução mínima:<br>. Gméd < 2mmHg	- Obstrução Discreta:<br>. Gméd 2-4mmHg	- Reintervenção<br>- Obstrução moderada ou importante:<br>. Gméd > 4mmHg
Veia em Cimitarra, tunelização para átrio esquerdo	- Nenhuma obstrução<br>- Obstrução mínima:<br>. Gméd < 2mmHg	- Obstrução Discreta:<br>. Gméd 2-4mmHg	- Reintervenção<br>- Obstrução moderada ou importante:<br>. Gméd > 4mmHg
Sistema de Condução	- Condução Normal<br>- Sem mudanças em relação ao pré-op.	- Condução Normal<br>- Sem mudanças em relação ao pré-op.	Necessidade de marcapasso definitivo

Gméd=gradiente médio

Correção de Comunicação Interatrial tipo Seio Venoso

**Subprocedimentos**	**Classe 1**	**Classe 2**	**Classe 3**
Tunelização da veia pulmonar anômala	- Nenhuma obstrução de veia pulmonar<br>- Nenhuma obstrução de VCS ou VCI<br>- Obstrução mínima:<br>. Gméd < 2mmHg	- Obstrução Discreta:<br>. Gméd 2-4mmHg	- Reintervenção<br>- Obstrução moderada ou importante:<br>. Gméd > 4mmHg
a. VCS (em defeitos do seio venoso superior)<br><br>b. VCI (em defeitos do seio venoso inferior)	- Nenhuma obstrução de VCS<br>- Obstrução mínima:<br>. Gméd < 2mmHg<br>- Nenhuma obstrução de VCI<br>- Obstrução mínima:<br>. Gméd < 2mmHg	- Obstrução discreta de VCS ou VCI:<br>. Gméd 2-4mmHg<br>- Obstrução discreta de VCI:<br>. Gméd 2-4mmHg	- Reintervenção<br>- Obstrução > discreta de VCS:<br>. Gméd > 4mmHg<br>- Reintervenção<br>- Obstrução > discreta de VCS:<br>. Gméd > 4mmHg
Correção da CIA	- Sem shunt ou shunt mínimo:<br>. < 2mm se > 10kg<br>. < 1mm se < 10kg	- Shunt residual pequeno:<br>. 2-3mm se > 10kg<br>. 1-2mm se < 10kg	- Reintervenção<br>- Shunt residual moderado ou grande:<br>. > 3mm se > 10kg<br>. > 2mm se < 10kg
Sistema de Condução	- Condução Normal<br>- Sem mudanças em relação ao pré-op.	- Condução Normal<br>- Sem mudanças em relação ao pré-op.	Necessidade de marcapasso definitivo

Quando existe a intenção de fechamento parcial da CIA, a correção da CIA não é pontuada. Gméd=gradiente médio

Correção de Comunicação Interventricular (CIV), Ventriculoseptoplastia, Ventriculoseptorrafia, múltiplas

**Subprocedimentos**	**Classe 1**	**Classe 2**	**Classe 3**
Correção da CIA	- Sem shunt ou shunt mínimo:<br>. < 2mm se > 10kg<br>. < 1mm se < 10kg	- Shunt residual pequeno:<br>. 2-3mm se > 10kg<br>. 1-2mm se < 10kg	- Reintervenção<br>- Shunt residual moderado ou grande:<br>. > 3mm se > 10kg<br>. > 2mm se < 10kg
Correção CIV, perimembranosa	- Sem shunt ou shunt mínimo:<br>. < 2mm se > 10kg<br>. < 1mm se < 10kg	- Shunt residual pequeno:<br>. 2-3mm se > 10kg<br>. 1-2mm se < 10kg	- Reintervenção<br>- Shunt residual moderado ou grande:<br>. > 3mm se > 10kg<br>. > 2mm se < 10kg
Correção CIV, muscular	- Sem shunt ou shunt mínimo:<br>. < 2mm se > 10kg<br>. < 1mm se < 10kg	- Shunt residual pequeno:<br>. 2-3mm se > 10kg<br>. 1-2mm se < 10kg	- Reintervenção<br>- Shunt residual moderado ou grande:<br>. > 3mm se > 10kg<br>. > 2mm se < 10kg
Ligadura/Secção PCA	- Sem shunt residual	- Shunt residual < 1mm	- Reintervenção<br>- Shunt > 1mm
Sistema de Condução	- Condução Normal<br>- Sem mudanças em relação ao pré-op.	- Condução Normal<br>- Sem mudanças em relação ao pré-op.	Necessidade de marcapasso definitivo

Quando existe a intenção de fechamento parcial da CIA, a correção da CIA não é pontuada.
Quando existe a intenção de fechamento de CIV com fenestração, a correção da CIV não é pontuada.
Outras CIVs não abordadas cirurgicamente não são pontuadas.

Correção de DSAV (Parcial, Transicional, Total)

**Subprocedimentos**	**Classe 1**	**Classe 2**	**Classe 3**
Correção da CIA, ostium primum	- Sem shunt ou shunt mínimo:<br>. < 2mm se > 10kg<br>. < 1mm se < 10kg	- Shunt residual pequeno:<br>. 2-3mm se > 10kg<br>. 1-2mm se < 10kg	- Reintervenção<br>- Shunt residual moderado ou grande:<br>. > 3mm se > 10kg<br>. > 2mm se < 10kg
Correção da CIA, ostium secundum	- Sem shunt ou shunt mínimo:<br>. < 2mm se > 10kg<br>. < 1mm se < 10kg	- Shunt residual pequeno:<br>. 2-3mm se > 10kg<br>. 1-2mm se < 10kg	- Reintervenção<br>- Shunt residual moderado ou grande:<br>. > 3mm se > 10kg<br>. > 2mm se < 10kg
Correção da CIV	- Sem shunt ou shunt mínimo:<br>. < 2mm se > 10kg<br>. < 1mm se < 10kg	- Shunt residual pequeno:<br>. 2-3mm se > 10kg<br>. 1-2mm se < 10kg	- Reintervenção<br>- Shunt residual moderado ou grande:<br>. > 3mm se > 10kg<br>. > 2mm se < 10kg
Plastia de Valva AV Esquerda	- Nenhuma Estenose<br>- Estenose mínima:<br>. Gméd ≤ 3mHg<br>- Nenhuma Insuficiência<br>- Insuficiência mínima:<br>. VC < 1 mm se < 10kg<br>. VC < 2 mm se > 10kg	- Estenose Discreta:<br>. Gméd 3-6mmHg<br>- Insuficiência Discreta:<br>. VC 1-2mm se < 10kg<br>. VC 2-4mm se > 10kg	- Reintervenção<br>- Estenose moderada ou importante:<br>. Gméd > 6mmHg<br>- Insuficiência moderada ou importante:<br>. VC > 2mm, se < 10kg<br>. VC > 4mm, se > 10kg
Plastia de Valva AV direita	- Nenhuma Estenose<br>- Estenose mínima:<br>. Gméd ≤ 3mHg<br>- Nenhuma Insuficiência<br>- Insuficiência mínima:<br>. VC < 3 mm se < 10kg<br>. VC < 4 mm se > 10kg	- Estenose Discreta:<br>. Gméd 3-6mmHg<br>- Insuficiência Discreta:<br>. VC 3-5mm se < 10kg<br>. VC 4-6mm se > 10kg	- Reintervenção<br>- Estenose moderada ou importante:<br>. Gméd > 6mmHg<br>- Insuficiência moderada ou importante:<br>. VC > 5mm, se < 10kg<br>. VC > 6mm, se > 10kg
Via de saída do VE (VSVE)	- Nenhuma Obstrução na VSVE<br>- Obstrução mínima:<br>. Gmáx < 20mmHg	- Obstrução VSVE discreta:<br>. Gmáx 20-40mHg	- Reintervenção<br>- Obstrução VSVE moderada ou importante:<br>. Gmáx > 40mmHg
Sistema de Condução	- Condução Normal<br>- Sem mudanças em relação ao pré-op.	- Condução Normal<br>- Sem mudanças em relação ao pré-op.	Necessidade de marcapasso definitivo

Gmáx=gradiente máximo, Gméd=gradiente médio,VC=vena contracta
Atenção: Gradiente máximo (Gmáx) ou Gradiente de pico instantâneo (MIG) é usado para Obstrução VSVE e para os gradientes da Valva Aórtica.
Não existe critério de peso para lesões estenóticas. Estratificações de peso (ex.: < 10kg e > 10kg) são usadas apenas para insuficiências.
Quando existe a intenção de fechamento parcial da CIA, a correção da CIA não é pontuada.

Correção de Tetralogia de Fallot – T4F (+/- ventriculotomia, +/- remendo transanular, preservação da valva pulmonar)

Subprocedimentos	Classe 1	Classe 2	Classe 3
Correção da CIA, ostium secundum	- Sem shunt ou shunt mínimo:<br>. < 2mm se > 10kg<br>. < 1mm se < 10kg	- Shunt residual pequeno:<br>. 2-3mm se > 10kg<br>. 1-2mm se < 10kg	- Reintervenção<br>- Shunt residual moderado ou grande:<br>. > 3mm se > 10kg<br>. > 2mm se < 10kg
Correção da CIV, conoventricular	- Sem shunt ou shunt mínimo:<br>. < 2mm se > 10kg<br>. < 1mm se < 10kg	- Shunt residual pequeno:<br>. 2-3mm se > 10kg<br>. 1-2mm se < 10kg	- Reintervenção<br>- Shunt residual moderado ou grande:<br>. > 3mm se > 10kg<br>. > 2mm se < 10kg
Correção CIV, muscular	- Sem shunt ou shunt mínimo:<br>. < 2mm se > 10kg<br>. < 1mm se < 10kg	- Shunt residual pequeno:<br>. 2-3mm se > 10kg<br>. 1-2mm se < 10kg	- Reintervenção<br>- Shunt residual moderado ou grande:<br>. > 3mm se > 10kg<br>. > 2mm se < 10kg
Via de saída do VD (VSVD)	- Nenhuma Obstrução na VSVE<br>- Obstrução mínima:<br>. Gmáx < 20mmHg	- Obstrução VSVE discreta:<br>. Gmáx 20-40mHg	- Reintervenção<br>- Obstrução VSVE moderada ou importante:<br>. Gmáx > 40mmHg
Reconstrução da Valva Pulmonar<br>- casos com preservação da valva pulmonar	- VSVD Gmáx < 20mmHg<br>- Nenhuma Insuficiência Pulmonar<br>- Insuficiência Pulmonar Discreta:<br>. VC < 3mm se < 10kg<br>. VC < 5mm se > 10 kg	- VSVD Gmáx 20-40mmHg<br>- Insuficiência Pulmonar Discreta:<br>. VC 3-5mm se < 10kg<br>. VC 5-8mm se > 10kg	- VSVD Gmáx > 40mmHg<br>- Insuficiência Pulmonar Moderada ou Importante:<br>. VC > 5mm se < 10kg<br>. VC > 8mm se> 10kg
Reconstrução da Valva Pulmonar<br>- casos com patch transanular	- VSVD Gmáx < 20mmHg<br>-Insuficiência Pulmonar:<br>. Sem consequência	- VSVD Gmáx 20-40mmHg<br>- Insuficiência Pulmonar:<br>. Sem consequência	- VSVD Gmáx > 40mmHg<br>- Insuficiência Pulmonar:<br>. Sem consequência
Reconstrução do Tronco Pulmonar	- Nenhuma Obstrução ou estreitamento:<br>. Gmáx < 10mmHg	- Estreitamento discreto<br>- Obstrução discreta:<br>. Gmáx 10-20mHg	- Reintervenção<br>- Obstrução moderada ou importante:<br>. Gmáx > 20mmHg
Reconstrução da Artéria Pulmonar Direita	- Nenhuma Obstrução ou estreitamento:<br>. Gmáx < 20mmHg<br>- Nenhum estreitamento em exames de imagem ou no doppler colorido	- Estreitamento discreto com gradiente residual discreto:<br>. Gmáx 20-40mmHg<br>- Estreitamento < 30% em exames de imagem ou no doppler colorido	- Reintervenção<br>- Gmáx > 40mmHg<br>- > 30% de estreitamento em exames de imagem ou no doppler colorido
Reconstrução da Artéria Pulmonar Esquerda	- Nenhuma Obstrução ou estreitamento:<br>. Gmáx < 20mmHg<br>- Nenhum estreitamento em exames de imagem ou doppler colorido	- Estreitamento discreto com gradiente residual discreto:<br>. Gmáx 20-40mmHg<br>- Estreitamento < 30% em exames de imagem ou na largura do jato do doppler colorido	- Reintervenção<br>- Gmáx > 40mmHg<br>- > 30% de estreitamento em exames de imagem ou na largura do jato do doppler colorido
Ligadura/Secção PCA	- Sem shunt residual	- Shunt residual < 1mm	- Reintervenção<br>- Shunt > 1mm
Sistema de Condução	- Condução Normal<br>- Sem mudanças em relação ao pré-op.	- Condução Normal<br>- Sem mudanças em relação ao pré-op.	Necessidade de marcapasso definitivo

Quando existe a intenção de fechamento parcial da CIA, a correção da CIA não é pontuada.
Quando existe a intenção de fechamento de CIV com fenestração, a correção da CIV não é pontuada.
Gmáx=gradiente máximo, VC=vena contracta

Correção de Coarctação da Aorta – CoAo (término-terminal, término-terminal extendida, +/- plastia com retalho ou remendo, +/- interposição de enxerto

**Subprocedimentos**	**Classe 1**	**Classe 2**	**Classe 3**
Aorta Descendente	- Nenhum gradiente residual<br>- Gradiente mínimo:<br>. Gmáx < 20mmHg	- Estenose discreta:<br>. Gmáx 20-40mmHg	- Reintervenção<br>-Estenose moderada ou importante:<br>. Gmáx > 40mmHg
Arco Aórtico	- Nenhuma Obstrução ou estreitamento:<br>. Gmáx < 20mmHg<br>- Nenhum estreitamento em exames de imagem ou doppler colorido	- Estreitamento discreto com gradiente residual discreto:<br>. Gmáx 20-40mmHg<br>- Estreitamento < 30% em exames de imagem ou na largura do jato do doppler colorido	- Reintervenção<br>- Gmáx > 40mmHg<br>- > 30% de estreitamento em exames de imagem ou na largura do jato do doppler colorido

Gmáx=gradiente máximo

GRUPO 2

Operação de Jatene (+ variações de D-TGA/septo interventricular intacto, D-TGA/CIV, D-TGA/+/- CIV/anomalia do arco aórtico, Taussig-Bing)

Subprocedimentos	Classe 1	Classe 2	Classe 3
Correção da CIA, ostium secundum	- Sem shunt ou shunt mínimo:<br>. < 2mm se > 10kg<br>. < 1mm se < 10kg	- Shunt residual pequeno:<br>. 2-3mm se > 10kg<br>. 1-2mm se < 10kg	- Reintervenção<br>- Shunt residual moderado ou grande:<br>. > 3mm se > 10kg<br>. > 2mm se < 10kg
Correção da CIV	- Sem shunt ou shunt mínimo:<br>. < 2mm se > 10kg<br>. < 1mm se < 10kg	- Shunt residual pequeno:<br>. 2-3mm se > 10kg<br>. 1-2mm se < 10kg	- Reintervenção<br>- Shunt residual moderado ou grande:<br>. > 3mm se > 10kg<br>. > 2mm se < 10kg
Correção da CIV, muscular	- Sem shunt ou shunt mínimo:<br>. < 2mm se > 10kg<br>. < 1mm se < 10kg	- Shunt residual pequeno:<br>. 2-3mm se > 10kg<br>. 1-2mm se < 10kg	- Reintervenção<br>- Shunt residual moderado ou grande:<br>. > 3mm se > 10kg<br>. > 2mm se < 10kg
Anastomose Suprapulmonar	- Nenhuma Obstrução ou estreitamento:<br>. Gmáx < 10mmHg	- Estreitamento discreto<br>- Obstrução discreta:<br>. Gmáx 10-20mHg	- Reintervenção<br>- Obstrução moderada ou importante:<br>. Gmáx > 20mmHg
Reconstrução de tronco e artérias pulmonares (plastia)	- Nenhuma Obstrução ou estreitamento:<br>. Gmáx < 20mmHg<br>- Nenhum estreitamento em exames de imagem ou doppler colorido	- Estreitamento discreto com gradiente residual discreto:<br>. Gmáx 20-40mmHg<br>- Estreitamento < 30% em exames de imagem ou na largura do jato do doppler colorido	- Reintervenção<br>- Obstrução moderada ou importante: Gmáx > 40mmHg<br>- > 30% de estreitamento em exames de imagem ou na largura do jato do doppler colorido
Anastomose Supra Aórtica	- Nenhuma Obstrução ou estreitamento:<br>. Gmáx < 10mmHg	- Estreitamento discreto<br>- Obstrução discreta:<br>. Gmáx 10-20mHg	- Reintervenção<br>- Obstrução moderada ou importante:<br>. Gmáx > 20mmHg
Via de saída sub pulmonar ou sub aórtica (quando abordada)	- Nenhum estreitamento ou gradiente residual<br>- Obstrução mínima:<br>. Gmáx < 20mmHg	- Estreitamento discreto<br>- Obstrução residual discreta:<br>. Gmáx 20-40mHg	- Reintervenção<br>- Obstrução moderada ou importante:<br>. Gmáx > 40mmHg
Reimplante de Coronárias	Sem obstrução ao fluxo coronariano	Sem obstrução ao fluxo coronariano	- Reintervenção<br>- Comprometimento do fluxo coronariano, isquemia/infarto, com alterações no ECO/ECG
Arco Aórtico (quando abordado)	- Nenhuma Obstrução ou estreitamento:<br>. Gmáx < 20mmHg<br>- Nenhum estreitamento em exames de imagem ou doppler colorido	- Estreitamento discreto com gradiente residual discreto:<br>. Gmáx 20-40mmHg<br>- Estreitamento < 30% em exames de imagem ou na largura do jato do doppler colorido	- Reintervenção<br>- Obstrução moderada ou importante<br>- Gmáx > 40mmHg<br>- > 30% de estreitamento em exames de imagem ou na largura do jato do doppler colorido
Sistema de Condução	- Condução Normal<br>- Sem mudanças em relação ao pré-op.	- Condução Normal<br>- Sem mudanças em relação ao pré-op.	Necessidade de marcapasso definitivo

Gmáx=gradiente máximo

Correção da Conexão Anômala Total de Veias Pulmonares – CATVP/DATVP (supra-cardíaca, intracardíaca, infra-cardíaca, mista)
Atenção: Os gradientes devem ser qualificados com base no débito cardíaco e/ou presença ou ausência de disfunção miocárdica

**Subprocedimentos**	**Classe 1**	**Classe 2**	**Classe 3**
Coletor ou tunelização das Veias Pulmonares	- Nenhuma obstrução de veia pulmonar<br>- Obstrução mínima:<br>. Gméd < 2mmHg	- Obstrução Discreta:<br>. Gméd: 2-4mmHg	- Reintervenção<br>- Obstrução moderada ou importante:<br>. Gméd: > 4mmHg
Abertura do teto do seio coronário	- Nenhuma obstrução de veia pulmonar<br>- Obstrução mínima:<br>. Gméd < 2mmHg	- Obstrução Discreta:<br>. Gméd: 2-4mmHg	- Reintervenção<br>- Obstrução moderada ou importante:<br>. Gméd: > 4mmHg
Correção da CIA, com remendo	- Sem shunt ou shunt mínimo:<br>. < 2mm se > 10kg<br>. < 1mm se < 10kg	- Shunt residual pequeno:<br>. 2-3mm se > 10kg<br>. 1-2mm se < 10kg	- Reintervenção<br>- Shunt residual moderado ou grande:<br>. > 3mm se > 10kg<br>. > 2mm se < 10kg
Drenagem Venosa Sistêmica	- Nenhuma obstrução<br>- Obstrução mínima:<br>. Gméd < 2mmHg	- Obstrução Discreta:<br>. Gméd: 2-4mmHg	- Reintervenção<br>- Obstrução moderada ou importante:<br>. Gméd: > 4mmHg
Sistema de Condução	- Condução Normal<br>- Sem mudanças em relação ao pré-op.	- Condução Normal<br>- Sem mudanças em relação ao pré-op.	Necessidade de marcapasso definitivo

Quando existe a intenção de fechamento parcial da CIA, a correção da CIA não é pontuada.
Gméd=gradiente médio

Correção da Interrupção do Arco Aórtico (IAAo) +/- CIV +/- CIA, Coarctação + CIV, Arco ártico +/- CIV

Subprocedimentos	Classe 1	Classe 2	Classe 3
Arco Aórtico	- Nenhuma Obstrução ou estreitamento:<br>. Gmáx < 20mmHg<br>- Nenhum estreitamento em exames de imagem ou doppler colorido	- Estreitamento discreto com gradiente residual discreto:<br>. Gmáx 20-40mmHg<br>- Estreitamento < 30% em exames de imagem ou na largura do jato do doppler colorido	- Reintervenção<br>- Obstrução moderada ou importante<br>- Gmáx > 40mmHg<br>- > 30% de estreitamento em exames de imagem ou na largura do jato do doppler colorido
Correção da CIV	- Sem shunt ou shunt mínimo:<br>. < 2mm se > 10kg<br>. < 1mm se < 10kg	- Shunt residual pequeno:<br>. 2-3mm se > 10kg<br>. 1-2mm se < 10kg	- Reintervenção<br>- Shunt residual moderado ou grande:<br>. > 3mm se > 10kg<br>. > 2mm se < 10kg
Correção da CIA, ostium secundum	- Sem shunt ou shunt mínimo:<br>. < 2mm se > 10kg<br>. < 1mm se < 10kg	- Shunt residual pequeno:<br>. 2-3mm se > 10kg<br>. 1-2mm se < 10kg	- Reintervenção<br>- Shunt residual moderado ou grande:<br>. > 3mm se > 10kg<br>. > 2mm se < 10kg
Via de saída do VE (VSVE)	- Nenhuma Obstrução na VSVE<br>- Obstrução mínima:<br>. Gmáx < 20mmHg	- Obstrução VSVE discreta:<br>. Gmáx 20-40mHg	- Reintervenção<br>- Obstrução VSVE moderada ou importante:<br>. Gmáx > 40mmHg
Sistema de Condução	- Condução Normal<br>- Sem mudanças em relação ao pré-op.	- Condução Normal<br>- Sem mudanças em relação ao pré-op.	Necessidade de marcapasso definitivo

Quando existe a intenção de fechamento parcial da CIA, a correção da CIA não é pontuada.
Quando existe a intenção de fechamento de CIV com fenestração, a correção da CIV não é pontuada.
Gmáx=gradiente máximo

Correção de Truncus Arteriosus (+-Interrupção do Arco Aórtico)

Subprocedimentos	Classe 1	Classe 2	Classe 3
Valva Truncal<br>(quando plastia)	- Nenhuma Estenose<br>- Estenose mínima:<br>. Gmáx ≤ 20mHg<br>- Nenhuma Insuficiência<br>- Insuficiência mínima:<br>. VC < 1 mm se < 10kg<br>. VC < 2 mm se > 10kg	- Estenose Discreta:<br>. Gmáx 20-40mmHg<br>- Insuficiência Discreta:<br>. VC 1-2mm se < 10kg<br>. VC 2-4mm se > 10kg	- Reintervenção<br>- Estenose moderada ou importante:<br>. Gmáx > 40mmHg<br>- Insuficiência moderada ou importante:<br>. VC > 2mm, se <10kg<br>. VC > 4mm, se > 10kg
Anastomose Supravalvar pulmonar	- Nenhuma Obstrução ou estreitamento:<br>. Gmáx < 10mmHg	- Estreitamento discreto<br>- Obstrução discreta:<br>. Gmáx 10-20mHg	- Reintervenção<br>- Obstrução moderada ou importante:<br>. Gmáx > 20mmHg
Tubo VD-TP/APs	- Nenhuma Obstrução ou estreitamento:<br>. Gmáx < 20mmHg<br>- Nenhum estreitamento em exames de imagem ou doppler colorido	- Estreitamento discreto com gradiente residual discreto:<br>. Gmáx 20-40mmHg<br>- Estreitamento < 30% em exames de imagem ou na largura do jato do doppler colorido	- Reintervenção<br>- Obstrução moderada ou importante<br>- Gmáx > 40mmHg<br>- > 30% de estreitamento em exames de imagem ou na largura do jato do doppler colorido
Anastomose Supravalvar Aórtica	- Nenhuma Obstrução ou estreitamento:<br>. Gmáx < 10mmHg	- Estreitamento discreto<br>- Obstrução discreta:<br>. Gmáx 10-20mHg	- Reintervenção<br>- Obstrução moderada ou importante:<br>. Gmáx > 20mmHg
Correção do Arco Aórtico/ Aorta Descendente	- Nenhuma Obstrução ou estreitamento:<br>. Gmáx < 20mmHg<br>- Nenhum estreitamento em exames de imagem ou doppler colorido	- Estreitamento discreto com gradiente residual discreto:<br>. Gmáx 20-40mmHg<br>- Estreitamento < 30% em exames de imagem ou na largura do jato do doppler colorido	- Reintervenção<br>- Obstrução moderada ou importante<br>- Gmáx > 40mmHg<br>- > 30% de estreitamento em exames de imagem ou na largura do jato do doppler colorido
Correção da CIV	- Sem shunt ou shunt mínimo:<br>. < 2mm se > 10kg<br>. < 1mm se < 10kg	- Shunt residual pequeno:<br>. 2-3mm se > 10kg<br>. 1-2mm se < 10kg	- Reintervenção<br>- Shunt residual moderado ou grande:<br>. > 3mm se > 10kg<br>. > 2mm se < 10kg
Correção da CIA, fechamento do FOP	- Sem shunt ou shunt mínimo:<br>. < 2mm se > 10kg<br>. < 1mm se < 10kg	- Shunt residual pequeno:<br>. 2-3mm se > 10kg<br>. 1-2mm se < 10kg	- Reintervenção<br>- Shunt residual moderado ou grande:<br>. > 3mm se > 10kg<br>. > 2mm se < 10kg
Via de saída do VE (VSVE), se abordada	- Nenhuma Obstrução na VSVE<br>- Obstrução mínima:<br>. Gmáx < 20mmHg	- Obstrução VSVE discreta:<br>. Gmáx 20-40mHg	- Reintervenção<br>- Obstrução VSVE moderada ou importante:<br>. Gmáx > 40mmHg
Sistema de Condução	- Condução Normal<br>- Sem mudanças em relação ao pré-op.	- Condução Normal<br>- Sem mudanças em relação ao pré-op.	Necessidade de marcapasso definitivo

Quando existe a intenção de fechamento parcial da CIA, a correção da CIA não é pontuada.
Quando existe a intenção de fechamento de CIV com fenestração, a correção da CIV não é pontuada
Gmáx=gradiente máximo, VC=vena contracta,

GRUPO 3

Shunt sistêmico-pulmonar, Operação de Blalock-Taussig Modificada

Subprocedimentos	Classe 1	Classe 2	Classe 3
Shunt/<br>Blalock-Taussig	Patente	Patente	- Reintervenção<br>-Oclusão parcial ou completa ou distorção de artérias pulmonares

Operação de Norwood

Subprocedimentos	Classe 1	Classe 2	Classe 3
Reconstrução do Arco Proximal	- Nenhuma Obstrução<br>- Nenhum estreitamento:<br>. Gmáx < 20mmHg<br>- Nenhum estreitamento em exames de imagem ou doppler colorido	- Estreitamento discreto com gradiente residual discreto:<br>. Gmáx 20-40mmHg<br>- Estreitamento < 30% em exames de imagem ou na largura do jato do doppler colorido	- Reintervenção<br>- Obstrução moderada ou importante<br>- Gmáx > 40mmHg<br>- > 30% de estreitamento em exames de imagem ou na largura do jato do doppler colorido
Reconstrução do Arco Distal	- Nenhuma Obstrução<br>- Nenhum estreitamento:<br>. Gmáx < 20mmHg<br>- Nenhum estreitamento em exames de imagem ou doppler colorido	- Estreitamento discreto com gradiente residual discreto:<br>. Gmáx 20-40mmHg<br>- Estreitamento < 30% em exames de imagem ou na largura do jato do doppler colorido	- Reintervenção<br>- Obstrução moderada ou importante<br>- Gmáx > 40mmHg<br>- > 30% de estreitamento em exames de imagem ou na largura do jato do doppler colorido
Perfusão coronariana	Sem obstrução ao fluxo nas artérias coronárias proximais	Sem obstrução ao fluxo nas artérias coronárias proximais	- Necessidade de reintervenção durante internação inicial<br>- Evidência de comprometimento do fluxo coronariano, isquemia/infarto, com alterações no ECO/ECG
Septectomia Atrial	- Nenhum gradiente residual<br>- Gradiente mínimo:<br>. Gméd < 2mmHg<br>(CIA restritiva propositalmente mantida - aceitar gradiente maior)	- Gradiente residual:<br>Gméd: 3-4mmHg (exceto se intencional)	- Necessidade de reintervenção<br>- Gméd > 4mmHg (exceto se intencional)
Fonte de fluxo Pulmonar			
a. Blalock-Taussig modificado	Patente	Patente	- Reintervenção<br>- Oclusão parcial ou completa ou distorção de artérias pulmonares
b.Tubo VD-APs (Shunt de Sano)<br>Correção da CIA, fechamento do FOP	Patente	Patente	- Reintervenção<br>- Oclusão parcial ou completa ou distorção de artérias pulmonares

Gmáx=gradiente máximo, Gméd=gradiente médio

Operação de Glenn (anastomose cavo-pulmonar uni- ou bidirecional; Glenn uni- ou bidirecional, +/- Reconstrução de artérias pulmonares)

Subprocedimentos	Classe 1	Classe 2	Classe 3
Anastomose de Glenn (VCS-AP)	- Nenhuma obstrução<br>- Nenhuma distorção de artérias pulmonares<br>. Gméd < 2mmHg	- Distorção discreta<br>- Obstrução discreta:<br>. Gméd 2-4mmHg	- Reintervenção<br>- Obstrução moderada ou importante:<br>. Gméd > 4mmHg
Sistema de Condução	- Condução Normal<br>- Sem mudanças em relação ao pré-op.	- Condução Normal<br>- Sem mudanças em relação ao pré-op.	Necessidade de marcapasso definitivo

Gméd=gradiente médio

Operação de Fontan, Derivação cavo-pulmonar total – CCPT (túnel lateral, +/- fenestração)

Subprocedimentos	Classe 1	Classe 2	Classe 3
Fluxo da VCI	- Nenhuma obstrução<br>- Nenhuma distorção de artérias pulmonares<br>. Gméd < 2mmHg	- Distorção discreta<br>- Obstrução discreta:<br>. Gméd 2-4mmHg	- Reintervenção<br>- Obstrução moderada ou importante:<br>. Gméd > 4mmHg
Anastomose Túnel-AP	- Nenhuma obstrução<br>- Nenhuma distorção de artérias pulmonares<br>. Gméd < 2mmHg	- Distorção discreta<br>- Obstrução discreta:<br>. Gméd 2-4mmHg	- Reintervenção<br>- Obstrução moderada ou importante:<br>. Gméd > 4mmHg
Fenestração (se realizada)	Patente	Patente	- Reintervenção<br>- Fenestração ocluída
Sistema de Condução	- Condução Normal<br>- Sem mudanças em relação ao pré-op.	- Condução Normal<br>- Sem mudanças em relação ao pré-op.	Necessidade de marcapasso definitivo

Gméd=gradiente médio

Operação de Fontan, Derivação cavo-pulmonar total – CCPT (tubo extra-cardíaco, +/- fenestração)

Subprocedimentos	Classe 1	Classe 2	Classe 3
Anastomose VCI-tubo extra	- Nenhuma obstrução<br>- Nenhuma distorção de artérias pulmonares<br>. Gméd < 2mmHg	- Distorção discreta<br>- Obstrução discreta:<br>. Gméd 2-4mmHg	- Reintervenção<br>- Obstrução moderada ou importante:<br>. Gméd > 4mmHg
Anastomose Tubo extra-AP	- Nenhuma obstrução<br>- Nenhuma distorção de artérias pulmonares<br>. Gméd < 2mmHg	- Distorção discreta<br>- Obstrução discreta:<br>. Gméd 2-4mmHg	- Reintervenção<br>- Obstrução moderada ou importante:<br>. Gméd > 4mmHg
Fenestração (se realizada)	Patente	Patente	- Reintervenção<br>- Fenestração ocluída
Sistema de Condução	- Condução Normal<br>- Sem mudanças em relação ao pré-op.	- Condução Normal<br>- Sem mudanças em relação ao pré-op.	Necessidade de marcapasso definitivo

Gméd=gradiente médio

GRUPO 4

Correção da Estenose Aórtica +/- Subvalvar +/- Supravalvar, +/- Valvar

Subprocedimentos	Classe 1	Classe 2	Classe 3
VSVE ressecção de membrana +/- miectomia	- Nenhuma estenose<br>- Estenose mínima:<br>. Gmáx < 20mmHg	- Estenose discreta:<br>. Gmáx 20-40mmHg	- Reintervenção<br>- Estenose moderada ou importante:<br>. Gmáx > 40mmHg
Valva Aórtica	- Nenhuma Estenose<br>- Estenose mínima:<br>. Gmáx ≤ 20mHg<br>- Nenhuma Insuficiência<br>- Insuficiência mínima:<br>. VC < 1 mm se < 10kg<br>. VC < 2 mm se > 10kg	- Estenose Discreta:<br>. Gmáx 20-40mmHg<br>- Insuficiência Discreta:<br>. VC 1-2mm se < 10kg<br>. VC 2-4mm se > 10kg	- Reintervenção<br>- Estenose moderada ou importante:<br>. Gmáx > 40mmHg<br>- Insuficiência moderada ou importante:<br>. VC > 2mm, se < 10kg<br>. VC > 4mm, se > 10kg
Fluxo coronariano	Sem obstrução ao fluxo coronariano	Sem obstrução ao fluxo coronariano	- Necessidade de reintervenção durante internação inicial<br>- Evidência de comprometimento do fluxo coronariano, isquemia/infarto, com alternações no ECO/ECG
Anastomose Supra-Aórtica	- Nenhuma Obstrução ou estreitamento:<br>. Gmáx < 10mmHg	- Estreitamento discreto<br>- Obstrução discreta:<br>. Gmáx 10-20mHg	- Reintervenção<br>- Obstrução moderada ou importante:<br>. Gmáx > 20mmHg
Correção do Arco Aórtico	- Nenhuma Obstrução<br>- Nenhum estreitamento:<br>. Gmáx < 20mmHg<br>- Nenhum estreitamento em exames de imagem ou doppler colorido	- Estreitamento discreto com gradiente residual discreto:<br>. Gmáx 20-40mmHg<br>- Estreitamento < 30% em exames de imagem ou na largura do jato do doppler colorido	- Reintervenção<br>- Obstrução moderada ou importante<br>- Gmáx > 40mmHg<br>- > 30% de estreitamento em exames de imagem ou na largura do jato do doppler colorido
Aorta Descendente	- Nenhuma Obstrução<br>- Nenhum estreitamento:<br>. Gmáx < 20mmHg	- Estreitamento discreto com gradiente residual discreto:<br>. Gmáx 20-40mmHg	- Reintervenção<br>- Obstrução moderada ou importante<br>- Gmáx > 40mmHg
Sistema de Condução	- Condução Normal<br>- Sem mudanças em relação ao pré-op.	- Condução Normal<br>- Sem mudanças em relação ao pré-op.	Necessidade de marcapasso definitivo

Gmáx=gradiente máximo, VC=vena contracta

Troca Valvar Aórtica, incluindo Valva Truncal, Prótese mecânica, Prótese Biológica

Subprocedimentos	Classe 1	Classe 2	Classe 3
Implante de Prótese Valvar Aórtica	- Nenhuma estenose residual<br>- Estenose mínima:<br>. Velocidade de pico < 2,5 m/s<br>- Sem insuficiência<br>- Insuficiência mínima	- Estenose discreta:<br>. Velocidade de pico: entre 2,5 - 3,5 m/s<br>- Escape perivalvar discreto:<br>.VC < 2mm	- Reintervenção<br>- Estenose moderada ou importante:<br>. Velocidade de pico > 3,5 m/s<br>- Escape perivalvar significativo:<br>. VC > 2mm
Anastomose Supravalvar Aórtica	- Nenhuma Obstrução ou estreitamento:<br>. Gmáx < 10mmHg	- Estreitamento discreto<br>- Obstrução discreta:<br>. Gmáx 10-20mHg	- Reintervenção<br>- Obstrução moderada ou importante:<br>. Gmáx > 20mmHg
Fluxo coronariano	Sem obstrução ao fluxo coronariano	Sem obstrução ao fluxo coronariano	- Reintervenção<br>- Evidência de comprometimento do fluxo coronariano, isquemia/infarto
Sistema de Condução	- Condução Normal<br>- Sem mudanças em relação ao pré-op.	- Condução Normal<br>- Sem mudanças em relação ao pré-op.	Necessidade de marcapasso definitivo

Gmáx=gradiente máximo, VC=vena contracta

Operação de Ross, Ross-Konno, Konno modificado

Subprocedimentos	Classe 1	Classe 2	Classe 3
Implante de Autoenxerto	- Nenhuma estenose aórtica valvar<br>- Nenhuma insuficiência aórtica valvar	- Estenose mínima ou discreta:<br>. Gmáx < 30mmHg<br>- Insuficiência mínima ou discreta:<br>. VC 2-4mm	- Reintervenção<br>- Estenose moderada ou importante:<br>. Gmáx > 30mmHg<br>- Insuficiência moderada ou importante:<br>. VC > 4mm
Fluxo coronariano	Sem obstrução ao fluxo coronariano	Sem obstrução ao fluxo coronariano	- Reintervenção<br>- Evidência de comprometimento do fluxo coronariano, isquemia/infarto
Anastomose Supravalvar Aórtica	- Nenhuma Obstrução ou estreitamento:<br>. Gmáx < 10mmHg	- Estreitamento discreto<br>- Obstrução discreta:<br>. Gmáx 10-20mHg	- Reintervenção<br>- Obstrução moderada ou importante:<br>. Gmáx > 20mmHg
Tubo VD-TP/APs	- Nenhuma Estenose<br>- Estenose Mínima:<br>. Gmáx < 20mmHg<br>- Nenhuma insuficiência<br>- Insuficiência mínima:<br>. VC < 3mm<br>- Nenhuma distorção/ estenose de artérias pulmonares<br>- Distorção/Estenose mínima de artérias pulmonares:<br>. Gmáx < 20mmHg	- Gradiente residual discreto:<br>. Gmáx 20-40mmHg<br>- Insuficiência Discreta:<br>. VC 3-5 mm	- Reintervenção<br>- Estenose moderada ou importante:<br>. Gmáx > 40mmHg<br>- Insuficiência moderada ou importante:<br>.VC > 5mmHg
VSVE	- Nenhuma Obstrução ou estreitamento:<br>. Gmáx < 20mmHg	- Estreitamento discreto<br>- Obstrução discreta:<br>. Gmáx 20-40mHg	- Reintervenção<br>- Obstrução moderada ou importante:<br>. Gmáx > 40mmHg
Sistema de Condução	- Condução Normal<br>- Sem mudanças em relação ao pré-op.	- Condução Normal<br>- Sem mudanças em relação ao pré-op.	Necessidade de marcapasso definitivo

Gmáx=gradiente máximo, VC=vena contracta

Substituição da Raiz Aórtica, preservando a Valva ou homoenxerto

Subprocedimentos	Classe 1	Classe 2	Classe 3
Valva Aórtica	- Nenhuma Estenose<br>- Estenose mínima:<br>. Gmáx ≤ 20mHg<br>- Nenhuma Insuficiência<br>- Insuficiência mínima:<br>. VC < 1 mm se < 10kg<br>. VC < 2 mm se > 10kg	- Estenose Discreta:<br>. Gmáx 20-40mmHg<br>- Insuficiência Discreta:<br>. VC 1-2mm se < 10kg<br>. VC 2-4mm se > 10kg	- Reintervenção<br>- Estenose moderada ou importante:<br>. Gmáx > 40mmHg<br>- Insuficiência moderada ou importante:<br>. VC > 2mm, se < 10kg<br>. VC > 4mm, se > 10kg
Anastomose Supravalvar Aórtica	- Nenhuma Obstrução ou estreitamento:<br>. Gmáx < 10mmHg	- Estreitamento discreto<br>- Obstrução discreta:<br>. Gmáx 10-20mHg	- Reintervenção<br>- Obstrução moderada ou importante:<br>. Gmáx > 20mmHg
Fluxo coronariano	Sem obstrução ao fluxo coronariano	Sem obstrução ao fluxo coronariano	- Reintervenção<br>- Evidência de comprometimento do fluxo coronariano, isquemia/infarto
Sistema de Condução	- Condução Normal<br>- Sem mudanças em relação ao pré-op.	- Condução Normal<br>- Sem mudanças em relação ao pré-op.	Necessidade de marcapasso definitivo

Gmáx=gradiente máximo, VC=vena contracta

Valvoplastia Mitral para Estenose ou Insuficiência, Anel Supramitral, Substituição Valvar Mitral, Prótese Biológica, Prótese Mecânica

Subprocedimentos	Classe 1	Classe 2	Classe 3
Plastia Valvar Mitral	- Nenhuma Estenose<br>- Estenose mínima:<br>. Gméd ≤ 3mHg<br>- Nenhuma Insuficiência<br>- Insuficiência mínima:<br>. VC < 1 mm se < 10kg<br>. VC < 2 mm se > 10kg	- Estenose Discreta:<br>. Gméd 3-6mmHg<br>- Insuficiência Discreta:<br>. VC 1-2mm se < 10kg<br>. VC 2-4mm se > 10kg	- Reintervenção<br>- Estenose moderada ou importante:<br>. Gméd > 6mmHg<br>- Insuficiência moderada ou importante:<br>. VC > 2mm, se < 10kg<br>. VC > 4mm, se > 10kg
Implante Valvar Mitral	- Nenhuma estenose residual<br>- Estenose mínima:<br>. Velocidade de pico < 1,5 m/s<br>- Sem escape perivalvar<br>- Escape perivalvar mínimo	- Estenose discreta:<br>. Velocidade de pico 1,5-2,5 m/s<br>- Escape perivalvar discreto:<br>.VC < 2mm	- Reintervenção<br>- Estenose moderada ou importante:<br>. Velocidade de pico > 2,5 m/s<br>- Escape perivalvar significativo:<br>. VC > 2mm
Fluxo coronariano	Sem obstrução ao fluxo coronariano	Sem obstrução ao fluxo coronariano	- Reintervenção<br>- Evidência de comprometimento do fluxo coronariano, isquemia/infarto
Sistema de Condução	- Condução Normal<br>- Sem mudanças em relação ao pré-op.	- Condução Normal<br>- Sem mudanças em relação ao pré-op.	Necessidade de marcapasso definitivo

Gméd=gradiente médio, VC=vena contracta

Valvoplastia Aórtica, Valva Truncal, para Estenose ou Insuficiência

Subprocedimentos	Classe 1	Classe 2	Classe 3
Plastia da Valva Aórtica ou Truncal	- Nenhuma Estenose<br>- Estenose mínima:<br>. Gmáx ≤ 20mHg<br>- Nenhuma Insuficiência<br>- Insuficiência mínima:<br>. VC < 1 mm se < 10kg<br>. VC < 2 mm se > 10kg	- Estenose Discreta:<br>. Gmáx 20-40mmHg<br>- Insuficiência Discreta:<br>. VC 1-2mm se < 10kg<br>. VC 2-4mm se > 10kg	- Reintervenção<br>- Estenose moderada ou importante:<br>. Gmáx > 40mmHg<br>- Insuficiência moderada ou importante:<br>. VC > 2mm, se < 10kg<br>. VC > 4mm, se > 10kg
Anastomose Supravalvar Aórtica	- Nenhuma Obstrução ou estreitamento:<br>. Gmáx < 10mmHg	- Estreitamento discreto<br>- Obstrução discreta:<br>. Gmáx 10-20mHg	- Reintervenção<br>- Obstrução moderada ou importante:<br>. Gmáx > 20mmHg
VSVE	- Nenhuma Obstrução ou estreitamento:<br>. Gmáx < 20mmHg	- Estreitamento discreto<br>- Obstrução discreta:<br>. Gmáx 20-40mHg	- Reintervenção<br>- Obstrução moderada ou importante:<br>. Gmáx > 40mmHg
Fluxo coronariano	Sem obstrução ao fluxo coronariano	Sem obstrução ao fluxo coronariano	- Reintervenção<br>- Evidência de comprometimento do fluxo coronariano, isquemia/infarto
Sistema de Condução	- Condução Normal<br>- Sem mudanças em relação ao pré-op.	- Condução Normal<br>- Sem mudanças em relação ao pré-op.	Necessidade de marcapasso definitivo

Gmáx=gradiente máximo, VC=vena contracta

Valvoplastia Tricúspide

Subprocedimentos	Classe 1	Classe 2	Classe 3
Plastia da Valva Tricúspide	- Nenhuma Estenose<br>- Estenose mínima:<br>. Gméd ≤ 3mHg<br>- Nenhuma Insuficiência<br>- Insuficiência mínima:<br>. VC < 3 mm se < 10kg<br>. VC < 4 mm se > 10kg	- Estenose Discreta:<br>. Gméd 3-6mmHg<br>- Insuficiência Discreta:<br>. VC 3-5mm se < 10kg<br>. VC 4-6mm se > 10kg	- Reintervenção<br>- Estenose moderada ou importante:<br>. Gméd > 6mmHg<br>- Insuficiência moderada ou importante:<br>. VC > 5mm, se < 10kg<br>. VC > 6mm, se > 10kg
Fluxo coronariano	Sem obstrução ao fluxo coronariano	Sem obstrução ao fluxo coronariano	- Reintervenção<br>- Evidência de comprometimento do fluxo coronariano, isquemia/infarto
Sistema de Condução	- Condução Normal<br>- Sem mudanças em relação ao pré-op.	- Condução Normal<br>- Sem mudanças em relação ao pré-op.	Necessidade de marcapasso definitivo

Gméd=gradiente médio, VC=vena contracta

Substituição da Valva Tricúspide

Subprocedimentos	Classe 1	Classe 2	Classe 3
Implante Valvar Tricúspide	- Nenhuma estenose residual<br>- Estenose mínima:<br>. Velocidade de pico < 1,5 m/s<br>- Sem escape perivalvar<br>- Escape perivalvar mínimo	- Estenose discreta:<br>. Velocidade de pico entre 1,5-2,5 m/s<br>- Escape perivalvar discreto:<br>.VC < 2mm	- Reintervenção<br>- Estenose moderada ou importante:<br>. Velocidade de pico > 2,5 m/s<br>- Escape perivalvar significativo:<br>. VC > 2mm
Fluxo coronariano	Sem obstrução ao fluxo coronariano	Sem obstrução ao fluxo coronariano	- Reintervenção<br>- Evidência de comprometimento do fluxo coronariano, isquemia/infarto
Sistema de Condução	- Condução Normal<br>- Sem mudanças em relação ao pré-op.	- Condução Normal<br>- Sem mudanças em relação ao pré-op.	Necessidade de marcapasso definitivo

VC=vena contracta

Correção da Anomalia de Ebstein/Técnica do Cone

Subprocedimentos	Classe 1	Classe 2	Classe 3
Plastia da Valva Tricúspide (Ebstein)	- Nenhuma Estenose<br>- Estenose mínima:<br>. Gméd ≤ 3mHg<br>- Nenhuma Insuficiência<br>- Insuficiência mínima:<br>. VC < 3 mm se < 10kg<br>. VC < 4 mm se > 10kg	- Estenose Discreta:<br>. Gméd 3-6mmHg<br>- Insuficiência Discreta:<br>. VC 3-5mm se < 10kg<br>. VC 4-6mm se > 10kg	- Reintervenção<br>- Estenose moderada ou importante:<br>. Gméd > 6mmHg<br>- Insuficiência moderada ou importante:<br>. VC > 5mm, se < 10kg<br>. VC > 6mm, se > 10kg
Plicatura anel valvar Tricúspide	- Nenhuma deformidade anatômica relacionada à plicatura<br>- Sem lesão de artéria coronária	- Nenhuma alteração anatômica relacionada à plicatura<br>- Sem lesão de artéria coronária	- Reintervenção<br>- Deiscência<br>- Lesão de artéria coronária
Plicatura/Excisão da porção atrializada do VD	- Nenhuma deformidade anatômica relacionada à plicatura<br>- Sem lesão de artéria coronária	- Nenhuma alteração anatômica relacionada à plicatura<br>- Sem lesão de artéria coronária	- Reintervenção<br>- Deiscência<br>- Lesão de artéria coronária
Sistema de Condução	- Condução Normal<br>- Sem mudanças em relação ao pré-op.	- Condução Normal<br>- Sem mudanças em relação ao pré-op.	Necessidade de marcapasso definitivo

Gméd=gradiente médio, VC=vena contracta

Troca da Valva Pulmonar / Correção da obstrução da Via de Saída do Ventrículo Direito, Tubo Ventrículo Direito-Artérias Pulmonares +/- Tronco Pulmonar, Plastia de artérias pulmonares, reconstrução (plastia) do Tronco e ou artérias pulmonares, reoperação de Tubo Ventrículo Direito-Artérias Pulmonares

**Subprocedimentos**	**Classe 1**	**Classe 2**	**Classe 3**
Substituição Valva Pulmonar	- Nenhuma estenose residual<br>- Estenose mínima:<br>. Velocidade de pico < 2,5 m/s<br>- Sem insuficiência<br>- Insuficiência mínima	- Estenose discreta:<br>. Velocidade de pico < 3,5 m/s<br>- Escape perivalvar discreto:<br>.VC < 2mm	- Reintervenção<br>- Estenose moderada ou importante:<br>. Velocidade de pico > 3,5 m/s<br>- Escape perivalvar significativo:<br>. VC > 2mm
Tubo VD-TP/artérias pulmonares<br>VSVD	- Nenhuma Estenose<br>- Estenose Mínima:<br>. Gmáx < 20mmHg<br>- Nenhuma insuficiência<br>- Insuficiência mínima:<br>. VC < 3mm	- Gradiente residual discreto:<br>. Gmáx 20-40mmHg<br>- Insuficiência Discreta:<br>. VC 3-5 mm	- Reintervenção<br>- Estenose moderada ou importante:<br>. Gmáx > 40mmHg<br>- Insuficiência moderada ou importante:<br>.VC > 5mmHg
Anastomose Supravalvar Pulmonar	- Nenhuma Obstrução ou estreitamento:<br>. Gmáx < 10mmHg	- Estreitamento discreto<br>- Obstrução discreta:<br>. Gmáx 10-20mHg	- Reintervenção<br>- Obstrução moderada ou importante:<br>. Gmáx > 20mmHg
Artérias Pulmonares	- Nenhum estreitamento<br>- Nenhum Gradiente<br>- Gradiente residual mínimo:<br>. Gmáx < 20mmHg<br>- Nenhum estreitamento em exames de imagem ou doppler colorido	- Estreitamento discreto com gradiente residual discreto:<br>. Gmáx 20-40mmHg<br>- Estreitamento < 30% em exames de imagem ou na largura do jato do doppler colorido	- Reintervenção<br>- Obstrução moderada ou importante<br>- Gmáx > 40mmHg<br>- > 30% de estreitamento em exames de imagem ou na largura do jato do doppler colorido
Correção da CIA/FOP	- Sem shunt ou shunt mínimo:<br>. < 2mm se > 10kg<br>. < 1mm se < 10kg	- Shunt residual pequeno:<br>. 2-3mm se > 10kg<br>. 1-2mm se < 10kg	- Reintervenção<br>- Shunt residual:<br>. > 3mm se > 10kg<br>. > 2mm se < 10kg
Sistema de Condução	- Condução Normal<br>- Sem mudanças em relação ao pré-op.	- Condução Normal<br>- Sem mudanças em relação ao pré-op.	Necessidade de marcapasso definitivo

Gmáx=gradiente máximo, VC=vena contracta

Correção Biventricular da Síndrome de Hipoplasia do Coração Esquerdo

**Subprocedimentos**	**Classe 1**	**Classe 2**	**Classe 3**
Anastomose Supravalvar Aórtica	- Nenhuma Obstrução ou estreitamento:<br>. Gmáx < 10mmHg	- Estreitamento discreto<br>- Obstrução discreta:<br>. Gmáx 10-20mHg	- Reintervenção<br>- Obstrução moderada ou importante:<br>. Gmáx > 20mmHg
Correção do Arco Aórtico	- Nenhuma Obstrução<br>- Nenhum estreitamento:<br>. Gmáx < 20mmHg<br>- Nenhum estreitamento em exames de imagem ou doppler colorido	- Estreitamento discreto com gradiente residual discreto:<br>. Gmáx 20-40mmHg<br>- Estreitamento < 30% em exames de imagem ou na largura do jato do doppler colorido	- Reintervenção<br>- Obstrução moderada ou importante<br>- Gmáx > 40mmHg<br>- > 30% de estreitamento em exames de imagem ou na largura do jato do doppler colorido
Aorta Descendente	- Nenhuma Obstrução<br>- Nenhum estreitamento:<br>. Gmáx < 20mmHg	- Estreitamento discreto com gradiente residual discreto:<br>. Gmáx 20-40mmHg	- Reintervenção<br>- Obstrução moderada ou importante<br>- Gmáx > 40mmHg
Plastia da Valva Aórtica	- Nenhuma Estenose<br>- Estenose mínima:<br>. Gmáx ≤ 20mHg<br>- Nenhuma Insuficiência<br>- Insuficiência mínima:<br>. VC < 1 mm se < 10kg<br>. VC < 2 mm se > 10kg	- Estenose Discreta:<br>. Gmáx 20-40mmHg<br>- Insuficiência Discreta:<br>. VC 1-2mm se < 10kg<br>. VC 2-4mm se > 10kg	- Reintervenção<br>- Estenose moderada ou importante:<br>. Gmáx > 40mmHg<br>- Insuficiência moderada ou importante:<br>. VC > 2mm, se < 10kg<br>. VC > 4mm, se > 10kg
Tubo VD-TP/artérias pulmonares<br>VSVD	- Nenhuma Estenose<br>- Estenose Mínima:<br>. Gmáx < 20mmHg<br>- Nenhuma insuficiência<br>- Insuficiência mínima:<br>. VC < 3mm	- Gradiente residual discreto:<br>. Gmáx 20-40mmHg<br>- Insuficiência Discreta:<br>. VC 3-5 mm	- Reintervenção<br>- Estenose moderada ou importante:<br>. Gmáx > 40mmHg<br>- Insuficiência moderada ou importante:<br>.VC > 5mmHg
Anastomose Supravalvar Pulmonar	- Nenhuma Obstrução ou estreitamento:<br>. Gmáx < 10mmHg	- Estreitamento discreto<br>- Obstrução discreta:<br>. Gmáx 10-20mmHg	- Reintervenção<br>- Obstrução moderada ou importante:<br>. Gmáx > 20mmHg
Artérias Pulmonares	- Nenhum estreitamento<br>- Nenhum Gradiente<br>- Gradiente residual mínimo:<br>. Gmáx < 20mmHg<br>- Nenhum estreitamento em exames de imagem ou doppler colorido	- Estreitamento discreto com gradiente residual discreto:<br>. Gmáx 20-40mmHg<br>- Estreitamento < 30% em exames de imagem ou na largura do jato do doppler colorido	- Reintervenção<br>- Estreitamento moderado ou importante<br>- Gmáx > 40mmHg<br>- > 30% de estreitamento em exames de imagem ou na largura do jato do doppler colorido
Conexão VCS-AD	- Nenhuma obstrução<br>- Nenhuma distorção de artérias pulmonares<br>. Gméd < 2mmHg	- Distorção discreta<br>- Obstrução discreta:<br>. Gméd 2-4mmHg	- Reintervenção<br>- Obstrução moderada ou importante:<br>. Gméd > 4mmHg
Correção da CIA/FOP	- Sem shunt ou shunt mínimo:<br>. < 2mm se > 10kg<br>. < 1mm se < 10kg	- Shunt residual pequeno:<br>. 2-3mm se > 10kg<br>. 1-2mm se < 10kg	- Reintervenção<br>- Shunt residual moderado ou grande:<br>. > 3mm se > 10kg<br>. > 2mm se < 10kg
Fluxo coronariano	Sem obstrução ao fluxo coronariano	Sem obstrução ao fluxo coronariano	- Reintervenção<br>- Evidência de comprometimento do fluxo coronariano, isquemia/infarto
Sistema de Condução	- Condução Normal<br>- Sem mudanças em relação ao pré-op.	- Condução Normal<br>- Sem mudanças em relação ao pré-op.	Necessidade de marcapasso definitivo

Gmáx=gradiente máximo, Gméd=gradiente médio, VC=vena contracta

Transplante Cardíaco

Subprocedimentos	Classe 1	Classe 2	Classe 3
Anastomose do AE	- Nenhuma obstrução<br>- Obstrução mínima:<br>. Gméd 1-2mmHg	- Obstrução discreta:<br>. Gméd 3-4mmHg	- Reintervenção<br>- Obstrução moderada ou importante:<br>. Gméd > 4mmHg
Anastomose Aórtica	- Nenhuma obstrução<br>- Gradiente mínimo:<br>. Gmáx < 10mmHg	- Obstrução discreta:<br>. Gmáx 10-20mmHg	- Reintervenção<br>- Obstrução moderada ou importante:<br>. Gmáx > 20mmHg
Anastomose Artéria Pulmonar	- Nenhuma obstrução<br>- Gradiente mínimo:<br>. Gmáx < 10mmHg	- Obstrução discreta:<br>. Gmáx 10-20mmHg	- Reintervenção<br>- Obstrução moderada ou importante:<br>. Gmáx > 20mmHg
Anastomose do VCS e da VCI	- Nenhuma obstrução<br>- Obstrução mínima:<br>. Gméd < 2mmHg	- Obstrução discreta:<br>. Gméd 2-4mmHg	- Reintervenção<br>- Obstrução moderada ou importante:<br>. Gméd > 4mmHg
Valva Tricúspide	- Nenhuma insuficiência Tricúspide	- Insuficiência Tricúspide discreta	- Insuficiência Tricúspide moderada ou importante
Correção da CIA/FOP	- Sem shunt ou shunt mínimo:<br>. < 2mm se > 10kg<br>. < 1mm se < 10kg	- Shunt residual pequeno:<br>. 2-3mm se > 10kg<br>. 1-2mm se < 10kg	- Reintervenção<br>- Shunt residual:<br>. > 3mm se > 10kg<br>. > 2mm se < 10kg
Sistema de Condução	- Condução Normal<br>- Sem mudanças em relação ao pré-op.	- Condução Normal<br>- Sem mudanças em relação ao pré-op.	Necessidade de marcapasso definitivo

Gmáx=gradiente máximo, Gméd=gradiente médio

Implante de Dispositivo de Assistência Circulatória Mecânica

Subprocedimentos	Classe 1	Classe 2	Classe 3
Inserção da Cânula do Ventrículo Esquerdo/Átrio Esquerdo	- Nenhuma obstrução ao fluxo	- Obstrução discreta ao fluxo	- Reintervenção
Inserção da Cânula na Aorta Ascendente	- Nenhuma obstrução ao fluxo<br>- Gmáx < 10mmHg	- Obstrução discreta ao fluxo:<br>. Gmáx 10-20mmHg	- Reintervenção<br>- Obstrução ao fluxo moderada ou importante:<br>. Gmáx > 20mmHg
Inserção da Cânula na Artéria Pulmonar	- Nenhuma obstrução ao fluxo<br>- Gmáx < 10mmHg	- Obstrução discreta ao fluxo:<br>. Gmáx 10-20mmHg	- Reintervenção<br>- Obstrução ao fluxo moderada ou importante:<br>. Gmáx > 20mmHg
Inserção da Cânula no AD	- Nenhuma obstrução ao fluxo	Obstrução discreta ao fluxo	- Reintervenção<br>- Obstrução moderada ou importante ao fluxo

Gmáx=gradiente máximo

Glossário - Glossary

Termo em Inglês	Sigla em Inglês	Termo em Português	Sigla em Português
English term	*English Acronym*	*Portuguese term*	*Portuguese Acronym*
Patent ductus arteriosus	PDA	Persistência do Canal Arterial	PCA
Atrial Septal Defect	ASD	Comunicação Interatrial	CIA
Partial anomalous pulmonary venous connection	PAPVC	Conexão/Drenagem anômala parcial das veias pulmonares	CAPVP, DAPVP
Sinus venosus defect		CIA tipo seio venoso	
Superior Vena Cava	SVC	Veia Cava Superior	VCS
Inferior Vena Cava	IVC	Veia Cava Inferior	VCI
Ventricular Septal Defect	VSD	Comunicação Interventricular	CIV
Atrioventricular septal defect (Atrioventricular Canal)	AVSD (AVC)	Defeito do Septo Atrioventricular (Canal átrio-ventricular)	DSAV (Canal AV)
Tetralogy of Fallot	TOF	Tetralogia de Fallot	T4F
Arterial Switch Operation	ASO	Operação de Jatene	
Transposition of the Great Arteries	TGA	Transposição das Grandes Artérias	TGA
Intact Ventricular Septum	IVS	Septo Interventricular Íntegro	SI
Total anomalous pulmonary venous return	TAPVC	Drenagem anômala total das veias pulmonares	DATVP
Interrupt Aortic Arch	IAA	Interrupção do Arco Aórtico	IAAo
Modified Blalock Taussig Shunt	MBTS	Blalock Taussig Modificado	BTM
Pulmonary Artery	PA	Artéria Pulmonar	AP
Total cavopulmonary connection	TCPC	Conexão Cavopulmonar Total	CCPT
Aortic Valve Replacement	AVR	Troca da Valva Aórtica	TVAo
Mitral Valve Replacement	MVR	Troca da Valva Mitral	TVMi
Pulmonary Valve Replacement	PVR	Troca da Valva Pulmonar	TVP
Right Ventricle Outflow Tract Obstruction	RVOTO	Obstrução da Via de Saída do Ventrículo Direito	OVSVD
Left Ventricle Outflow Tract Obstruction	LVOTO	Obstrução da Via de Saída do Ventrículo Esquerdo	OVSVE
Hypoplastic left heart syndrome	HLHS	Síndrome de Hipoplasia do Coração Esquerdo	SHCE
Peak Gradient	Gmax or MIG	Gradiente Máximo	Gmáx
Mean Gradient	Gmean	Gradiente Médio	Gméd
Vena Contracta	VC	Vena Contracta	VC
